# Berries as Nature’s Therapeutics: Exploring the Potential of *Vaccinium* Metabolites in Gastric Cancer Treatment Through Computational Insights

**DOI:** 10.3390/life15030406

**Published:** 2025-03-05

**Authors:** Angelica Rachel Carpio, Nicholas Dale Talubo, Po-Wei Tsai, Bor-Yann Chen, Lemmuel L. Tayo

**Affiliations:** 1 School of Chemical, Biological, and Materials Engineering and Sciences, School of Graduate Studies, Mapúa University, Manila 1002, Philippines; arecarpio@mymail.mapua.edu.ph (A.R.C.); nddtalubo@mymail.mapua.edu.ph (N.D.T.); 2 Department of Food Science, National Taiwan Ocean University, Keelung 202, Taiwan; powei@mail.ntou.edu.tw; 3 Department of Chemical and Materials Engineering, National I-Lan University, I-Lan 260, Taiwan; 4 Department of Biology, School of Health Sciences, Mapúa University, Makati 1200, Philippines

**Keywords:** cyanidin 3-O-glucoside, gastric cancer, molecular docking, molecular dynamics, *Vaccinium* species

## Abstract

Berries from the *Vaccinium* genus, known for their rich array of bioactive metabolites, are recognized for their antioxidant, anti-inflammatory, and anticancer properties. These compounds, including anthocyanins, flavonoids, and phenolic acids, have attracted significant attention for their potential health benefits, particularly in cancer prevention and treatment. Gastric cancer (GC), a leading cause of cancer-related deaths worldwide, remains challenging to treat, especially in its advanced stages. This study investigates the therapeutic potential of *Vaccinium* species in GC treatment using computational methods. RNA sequencing revealed upregulated genes associated with GC, while network pharmacology and molecular docking approaches identified strong interactions between cyanidin 3-O-glucoside (C3G), a key bioactive metabolite. Furthermore, molecular dynamics simulations of the HSP90AA1-C3G complex demonstrated stable binding and structural integrity, suggesting that C3G may inhibit HSP90AA1, a protein involved in cancer progression. These findings highlight the therapeutic potential of *Vaccinium* metabolites, offering a novel approach to GC treatment by targeting key molecular pathways. This research provides valuable insights into the role of berries as natural therapeutics, supporting their integration into future gastric cancer treatment strategies.

## 1. Introduction

Berries are valuable natural resources, representing the intricate connection between ecosystems and biodiversity. Found in diverse environments, these berries are not only integral to plant biodiversity but are also essential for the sustenance of various animal species [1]. Animals consume berries as a source of nutrition, aiding in seed dispersal and fostering ecosystem resilience [2,3,4]. Berries, especially those belonging to the *Vaccinium* genus, contribute profoundly to health promotion through their abundant antioxidants, diverse flavonoids, and a wide range of other beneficial phytochemicals [5] that benefit not only the animals that consume them but also humans, making them a staple in diets and traditional medicine across cultures.

The *Vaccinium* genus includes around 450 species, with commercially important varieties such as northern highbush blueberry (*V. corymbosum* L.), rabbiteye blueberry (*V. virgatum*), lowbush blueberry (*V. angustifolium*), bilberry (*V. myrtillus* L.), cranberry (*V. macrocarpon*), and lingonberry (*V. vitis-idaea* L.) [6]. Rising consumer demand, driven by scientific interest, has significantly boosted the market for blueberries and cranberries [7]. Wild species also support ecosystem balance as essential food sources for wildlife [8].

The bioactive metabolites in *Vaccinium* species, particularly in their leaves, stems, and fruits, have been widely used in functional foods and traditional medicine. These compounds exhibit antidiabetic, anti-inflammatory, antioxidant, and anticancer properties, contributing to reduced inflammation, improved digestive and urinary health, and lower risks of cardiovascular disease, obesity, cancer, and type 2 diabetes [5,9,10,11]. Their health benefits are largely attributed to anthocyanins and flavonoids, which have gained attention in crop breeding [6]. Key bioactive compounds include anthocyanins (cyanidin, peonidin, petunidin, delphinidin, and malvidin), proanthocyanidins, flavonols (quercetin, kaempferol, and myricetin), flavanols (epicatechin), phenolic acids (gallic, p-coumaric, caffeic, and chlorogenic), and ursolic acid, many of which exhibit potential anticancer effects [12].

Building on their well-documented bioactive properties, *Vaccinium* species have demonstrated potential in gastric cancer prevention and treatment. This study explores their underlying mechanisms, highlighting key phytochemicals and their interactions with molecular targets involved in gastric carcinogenesis. Their therapeutic potential aligns with the growing interest in plant-derived compounds as complementary or alternative strategies in cancer therapy.

Gastric cancer (GC) is the fifth most common cancer and the fourth leading cause of cancer deaths, with 1.09 million cases and 769,000 deaths in 2020, primarily in Asia, Latin America, and Europe [13,14]. Standard treatments, including surgery, chemotherapy, radiotherapy, immunotherapy, and targeted therapy, are often combined, but survival remains poor, especially in advanced stages [15,16]. Molecular pathologies in GC involve oncogene activation, tumor suppressor gene inactivation, CpG island methylation, and DNA repair defects, influencing diagnosis and treatment. Molecular testing, such as CDH1 gene screening for hereditary diffuse gastric carcinoma (HDGC) and HER2 expression analysis, is now standard in clinical practice [17]. Plant-derived compounds have gained increasing attention in cancer therapy due to their bioactive properties, lower toxicity, and potential to complement conventional treatments. Medicinal herbs and their phytocompounds not only enhance survival and quality of life in cancer patients but also modulate immune function, which plays a critical role in cancer progression and treatment outcomes [18]. Notably, over half of cancer prescription drugs are derived from natural plant sources, emphasizing their essential role in drug development. These compounds can act as standalone therapeutic agents or be integrated with existing treatments to improve efficacy and reduce adverse effects [19]. Studies have demonstrated that phytochemicals can mitigate chemotherapy-induced toxicity, enhance immune responses, and potentially lower the risk of recurrence and metastasis [20,21,22]. Furthermore, plant-derived bioactive compounds—such as phytochemicals, minerals, and vitamins—have been shown to inhibit cancer cell proliferation and promote apoptotic cell death in various malignancies [23,24]. Given their diverse mechanisms of action, natural products continue to be a valuable resource for providing safer and more effective cancer therapies.

This study explores the therapeutic potential of *Vaccinium* species in the context of gastric cancer. Conducted entirely in silico, it integrates natural compounds with advanced computational methods such as RNA sequencing, network pharmacology, molecular docking, and molecular dynamics to identify how these bioactive metabolites target cancer-related pathways. Ultimately, this study advances the therapeutic potential of natural plant metabolites while promoting eco-friendly approaches in cancer treatment. It underscores the role of berries in health promotion and offers valuable insights into their potential as functional foods and natural therapeutics.

## 2. Materials and Methods

### 2.1. Collection, Screening, and ADMET-Based Filtering of Metabolites in the Vaccinium Genus

The National Center for Biotechnology Information (NCBI) PubChem Database (https://pubchem.ncbi.nlm.nih.gov, accessed on 11 June 2024) [25]; KNApSAcK: A Comprehensive Species-Metabolite Relationship Database (http://www.knapsackfamily.com/KNApSAcK/, accessed on 12 September 2024) [26]; and HMDB: The Human Metabolome Database (https://hmdb.ca/, accessed on 12 September 2024) [27] were utilized to identify metabolites present in the *Vaccinium* genus. These open-access chemistry databases enabled the retrieval of metabolites and their canonical SMILES. The SMILES were subsequently entered into ADMETLab 3.0 (https://admetmesh.scbdd.com/, accessed on 18 September 2024) [28] to filter the metabolites based on Lipinski’s rule and cell permeability (Caco-2).

### 2.2. Target Gene Prediction for the Vaccinium Genus

The potential target proteins of the bioactive metabolites were screened using SuperPred (SuperPred: update on drug classification and target prediction (https://prediction.charite.de/, accessed on 18 September 2024)) [29]. The canonical SMILES of the metabolites were entered into SuperPred to predict target proteins for *Vaccinium* genus, their probability scores where the structure binds with the specific target, and their target–gene interaction.

### 2.3. Collection of RNA Sequence Data Associated with Gastric Cancer

The RNA-Seq counts used in this study were retrieved and preprocessed using the R library recount3 version 1.16.0. The use of recount3 enabled the integration of multiple studies, as the data were processed in a uniform and annotation-agnostic manner [30]. Three RNA-seq studies were included in this analysis. They were queried through recount3 using the project IDs ERP010795, ERP010889, and STAD. When combined, the data provide a comprehensive view of the Correa Human Model of Gastric Carcinogenesis. Specifically, the data from project ERP010889, retrieved by recount3 from the European Nucleotide Archive, consist of RNA-seq from stomach tissue biopsies suffering from gastritis, atrophy, extensive atrophy, and intestinal metaplasia. Project ERP010795, also retrieved from the European Nucleotide Archive, includes data on low- and high-grade dysplasia, as well as early gastric cancer. Lastly, the project STAD was retrieved from The Cancer Genome Atlas (TCGA) Program. After retrieving and cleaning the count files and metadata, DESeq2 was used to identify upregulated genes for each condition. To further minimize batch effects and protocol variations, the normal samples available for each experiment were used as references for their corresponding diseased states. The standard DESeq2 workflow was followed, with a significance level set at an adjusted *p*-value of 0.05. Upregulated and downregulated genes were defined by a log fold change (logFC) greater than 1 or less than −1, respectively. The similarity between conditions was visualized using UpSet plots.

### 2.4. Compound–Protein and Protein–Protein Interaction Network Construction and Analysis

A compound–protein interaction network was created to visualize the relationship of the accepted compounds and the predicted targets. By considering the edges, the CPI of each condition was hierarchically clustered, grouping conditions with potential shared pathways and therapeutic compounds together. To achieve this, Compound–Gene pairs were created per CPI. A presence matrix was created for each string of Compound–Gene pairs, setting the values at either 0 or 1 for those absent or present, respectively. Afterwards, the presence matrix was converted into a distance matrix using the binary method in R command dist. With the distance matrix, hierarchical clustering was undertaken with the complete linkage method. The optimal number of clusters was then determined using the silhouette method. The number of clusters is based on the maximum silhouette score and was applied using the cutree function. The gene list of all conditions was combined together per cluster, and their protein–protein interaction was assessed through STRINGDb version 12.0 (15 October 2024). The gene symbols per list was input into the website and the species was set to homo sapiens. Furthermore, for the network type ’Full STRING Network’, a score cut-off of 0.4 and an FDR stringency of 5% was set as the parameters to construct the network. The networks per cluster were exported into Cytoscape 3.10.2 using the stringApp extension (version 2.1.1). The hub proteins of the PPI networks were identified using the topological algorithms provided by the Cytoscape extension cytoHubba (version 0.1). Moreover, the top five hub proteins per network were identified by ranking them with their degree, taking the highest scorers. The potential compounds that target the top degree proteins per cluster were then retrieved and used for further analysis.

### 2.5. Gene Ontology and Enrichment Analysis

Gene Ontology (GO) and Kyoto Encyclopedia of Genes and Genomes (KEGG) pathway enrichment analyses were performed to explore the biological mechanisms through which *Vaccinium* metabolites affect gastric cancer. Only human (“*Homo sapiens*”) data were utilized for these analyses. Common proteins identified from *Vaccinium* metabolites through SuperPred and gastric cancer RNA sequencing data were processed using the DAVID Functional Annotation Bioinformatics Microarray tool. This analysis determined GO terms related to biological processes (BPs), molecular functions (MFs), and cellular components (CCs), as well as KEGG pathways. KEGG serves as a comprehensive resource, integrating genomic and functional data that include drugs, genomes, enzymes, and biological pathways to systematically annotate proteins and model cellular processes. Furthermore, Reactome pathway analysis was conducted to identify significantly associated biological pathways (https://david.ncifcrf.gov/, accessed on 15 October 2024) [31,32]. The results were then visualized using SRPlot (https://www.bioinformatics.com.cn/en, accessed on 15 October 2024) [33], providing detailed graphical representations of the enriched GO terms and pathways, which helped clarify the biological processes involved.

### 2.6. Molecular Docking

Molecular docking was employed to evaluate the binding interactions between the target compounds from the *Vaccinium* genus and the hub target proteins identified in this study. The crystal structures of the human hub proteins were retrieved from the AlphaFold Protein Structure Database (https://alphafold.ebi.ac.uk, accessed on 20 September 2024) [34], while the 3D structures of the *Vaccinium* metabolites were obtained from the PubChem database (https://pubchem.ncbi.nlm.nih.gov/, accessed on 20 September 2024) [25]. Docking simulations were performed using CB-Dock 2 (https://cadd.labshare.cn/cb-dock2/index.php, accessed on 21 September 2024) [35], a specialized tool for protein–ligand docking that supports flexible receptor binding sites. This approach enabled a comprehensive assessment of potential interactions and binding affinities, offering insights into the efficacy of these metabolites in targeting stomach cancer-related proteins.

### 2.7. Molecular Dynamics Simulations

The protein–ligand molecular dynamics simulations were conducted using GROMACS version 2024.2, with CHAPERONg employed as an automated pipeline to streamline the simulation process. Local modifications were made to the pipeline to accommodate command changes introduced in the latest GROMACS version. The conventional molecular dynamics simulation pipeline in CHAPERONg was utilized. The protein–ligand complex, retrieved from the molecular docking results, was selected based on the highest binding efficiency among various ligand conformations and binding sites. Using PyMol version 3.0.0-6, the ligand and protein were separated from the PDB file. Hydrogen atoms were added to the ligand using Avogadro 2 version 1.99.0, and the structure was converted into the appropriate format. The simulations were performed in a cubic simulation box using the CHARMM36 force field (http://mackerell.umaryland.edu/charmm_ff.shtml#gromacs, accessed on 20 December 2024). The system was solvated with TIP3P water molecules, and a salt concentration of 0.1 mol/L was maintained. A constant temperature of 300 K was applied throughout the simulation, which ran for 15,000,000 steps (equivalent to 30 ns). Post-processing of simulation results was conducted using tools available in CHAPERONg version 1.0, and relevant plots were generated. Finally, md-davis version 0.4.1 was used to construct a hydrogen bond matrix for the protein–ligand complex, providing insights into the binding interactions.

## 3. Results

### 3.1. Bioactive Metabolites Present in Vaccinium Species 

Initially, 119 metabolites were identified and screened using the PubChem in Table S1, KNApSAcK, and HMDB databases. This list was refined to five key metabolites (Table 1) and selected based on Lipinski’s Rule of Five, Caco-2 permeability data from ADMETLab 2.0, and target prediction probabilities from SuperPred. These five metabolites are also known to have anticancer properties. For filtering, only metabolites with zero Lipinski’s rule violations were included—resveratrol, ursolic acid, scopoletin, and (+)-catechin—with the exception of cyanidin 3-O-glucoside (C3G), which was conditionally accepted due to its strong antioxidant activity, documented bioavailability, and therapeutic potential. The five identified metabolites demonstrate the potential to traverse intestinal cell membranes via passive diffusion, carrier-mediated uptake, or active transport. While resveratrol and scopoletin have Caco-2 values above −5.15, suggesting better-predicted permeability, the inclusion of metabolites with lower values accounts for the possibility of alternative absorption mechanisms beyond passive diffusion [36]. Table 1 lists all selected metabolites.

### 3.2. Predicted Target Proteins of Vaccinium Metabolites and Gastric Cancer 

The 119 metabolites from the *Vaccinium* genus were analyzed using SuperPred, applying a probability threshold of 60% (*p* > 0.60) to ensure reliable target predictions [37]; target proteins were then identified from gastric cancer RNA sequencing data.

**Figure 1:**
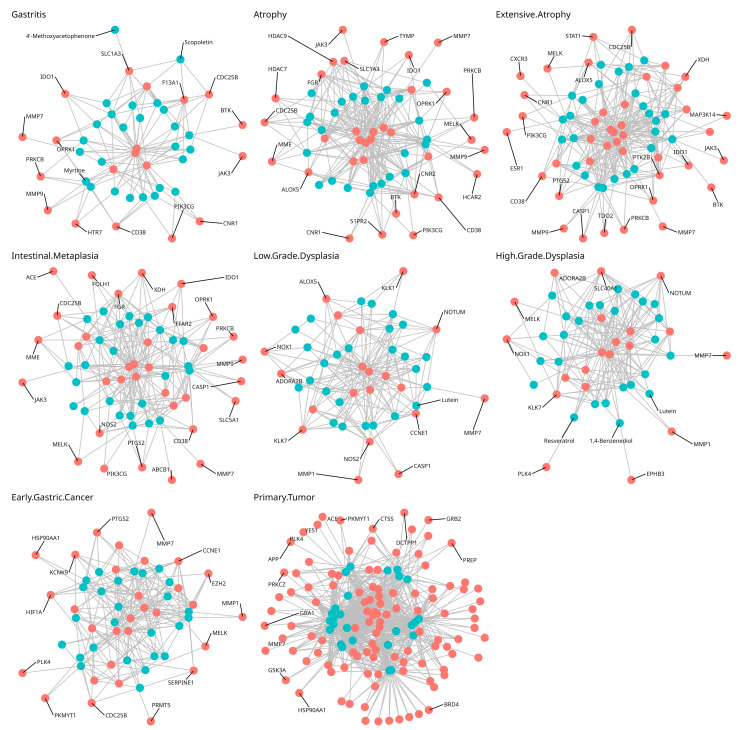
Compound–protein interaction maps for each stage in Correa’s cascade based on accepted *Vaccinium* metabolites and their predicted targets.

**Figure 2:**
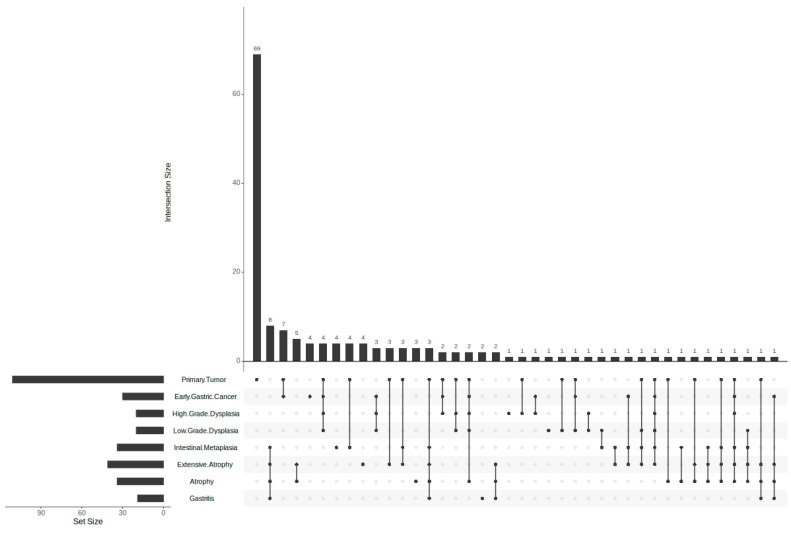
UpSet plot comparing the identified upregulated genes that interact with the examined metabolites.

Using the predicted targets of the identified metabolites, multiple compound–protein interaction (CPI) networks were constructed for the network pharmacology analysis. Figure 1 shows the CPI networks for each condition, with blue nodes representing compounds and red nodes representing proteins. As observed from above, the metabolites of the *Vaccinium* genus were predicted to potentially interact with the upregulated proteins of gastric cancer and precancerous lesions of the stomach. Interestingly, it could be observed that a significant jump in the number of upregulated genes happened in the gastric cancer samples (primary tumor). This indicates the increased dysregulation of the transcriptome as the process of the transformation from precancerous lesions to cancer is completed. Larger images of the network are available in Supplementary Figures S1–S8. Furthermore, a compound–pathway graph is available in Supplementary Figure S9. Figure 2 depicts the UpSet plot showing the increasing dysregulation of the transcriptome and the intersecting upregulated genes.

Given the focus on investigating the progression to gastric cancer, it is likely that the targets share therapeutic pathways. To explore these shared pathways and reduce the dimensionality of the data, hierarchical clustering was applied to a presence matrix of edges specific to each condition’s CPI. This approach identified similar networks, where selected metabolites target the same proteins, potentially indicating modulation across conditions. 

**Figure 3:**
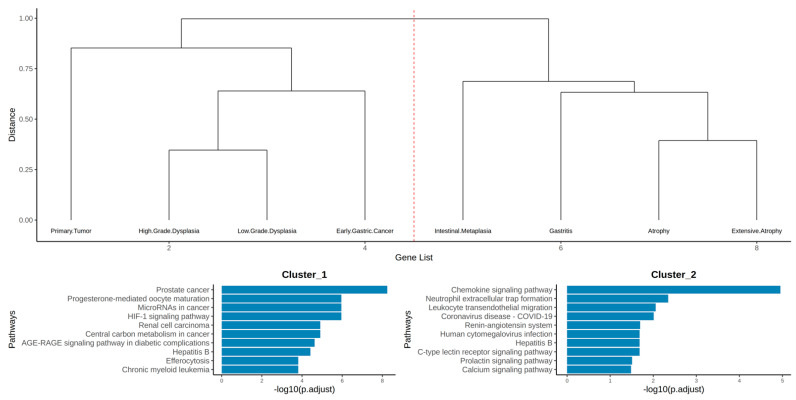
Hierarchical clustering results of CPI network edges with corresponding enrichment analysis of gene targets for each cluster. Cluster 1 is seen on the left side, while Cluster 2 is on the right side.

**Figure 4:**
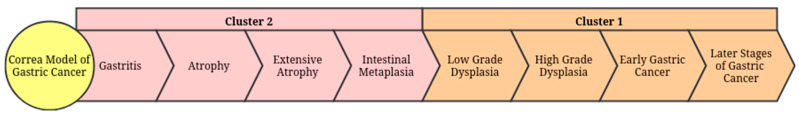
Model from RNA sequencing data showing histological progression toward gastric carcinogenesis, aligned with Correa’s cascade.

Figure 3 illustrates the hierarchical clustering of the edges in the CPI network. The number of clusters was computationally determined using the silhouette method. Two clusters were identified as ideal. Interestingly, these clusters appear to align with Correa’s cascade (see Figure 4), the histological progression model from active gastritis to gastric adenocarcinoma [38]. The computationally assigned Cluster 2 consists of the early stages of Correa’s cascade, beginning with gastritis and progressing through atrophy, extensive atrophy, and metaplasia. Cluster 1 completes Correa’s cascade, encompassing low-grade and high-grade dysplasia, early gastric cancer, and stomach adenocarcinoma. The biological relevance of this edge-based clustering is further supported by the gene enrichment results for each cluster. Cluster 1 was enriched in cancer-related terms, while Cluster 2 showed enrichment in immune system-related terms. 

**Figure 5:**
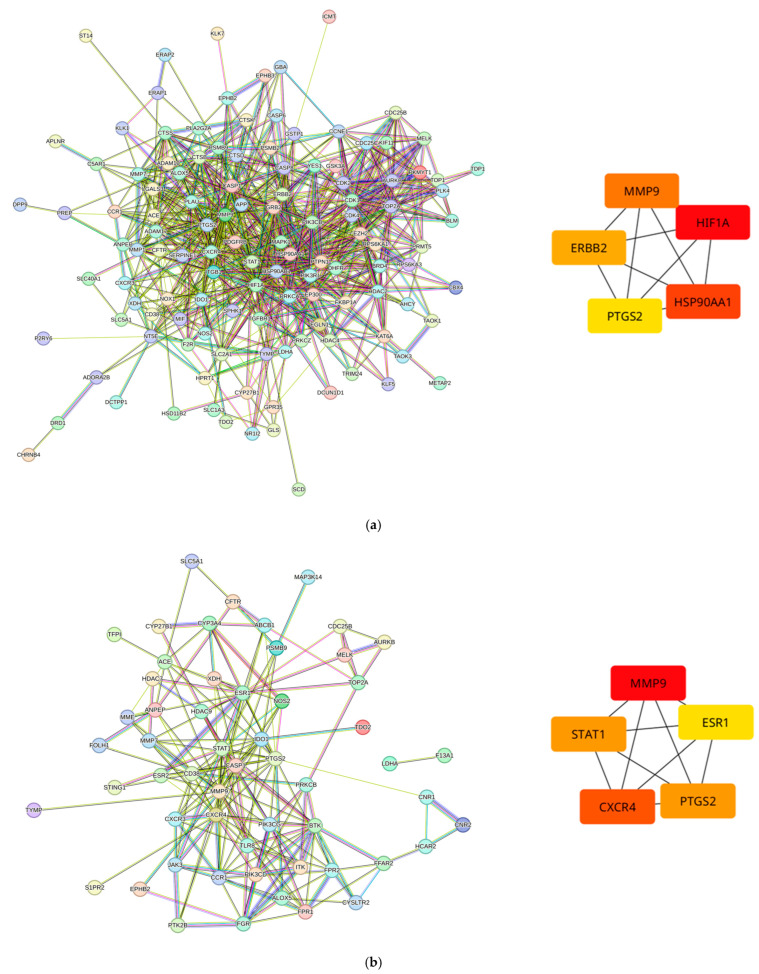
Generated protein–protein interaction (PPI) networks for (**a**) Cluster 1 and (**b**) Cluster 2 along with the top 5 hub proteins retrieved by Cytohubba.

In network pharmacology methodology, identifying high-impact proteins is essential to maximizing the theoretical potential of the compounds of interest. To achieve this, the gene list for each cluster was used to construct protein–protein interactions (PPIs), and their hub proteins were identified. By identifying proteins that are central to the network, the chances of selecting an essential and important protein in terms of biological function increases. The predicted compounds that potentially interact with these hub proteins were then considered compounds of interest. Figure 5 illustrates the PPI retrieved from STRINGdb, highlighting the top five hub proteins based on degree. As summarized in Table 2, Cluster 1 included the hub proteins MMP9, PTGS2, HIF1A, ERBB2, and HSP90AA1, while Cluster 2 comprised MMP9, PTGS2, CXCR4, ESR1, and STAT1. 

### 3.3. GO, KEGG, and Reactome Enrichment Analysis

**Figure 6:**
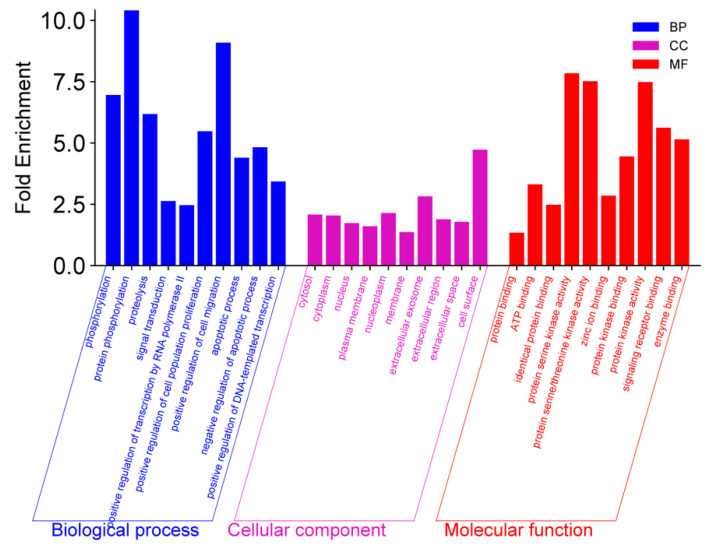
GO analysis and its three categories: biological process (BP), cellular component (CC), and molecular function (MF).

Enrichment analysis is essential for understanding the implications of intersecting proteins within complex biological systems and classifying gene functions through Gene Ontology (GO) terms, thereby facilitating hypothesis generation and pathway identification. This aims to identify biological annotations that are overrepresented in a gene list relative to a reference background [39]. Integrating GO analysis with additional bioinformatics tools improves the identification of new biomarkers and therapeutic targets for gastric cancer. Figure 6 illustrates the categories of biological processes (BPs), cellular components (CCs), and molecular functions (MFs). Within the biological processes, protein phosphorylation and positive regulation of cell migration emerged as significant contributors, exhibiting notable fold enrichment. In the cellular components, the fold enrichment values are relatively similar, with the cell surface demonstrating the highest enrichment, indicating a strong representation of proteins associated with membrane-bound processes. The molecular functions, on the other hand, highlight the significant emphasis on kinase activity and ATP binding, characterized by high fold enrichment and low FDR. Key terms such as protein serine kinase activity and protein kinase activity underscore the potential importance of signaling pathways and phosphorylation processes in gastric cancer.

**Figure 7:**
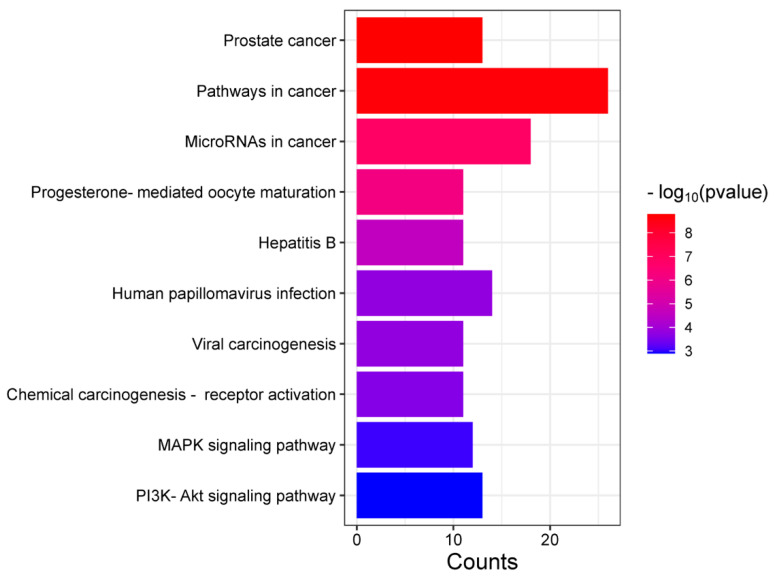
The KEGG pathway enrichment analysis is visualized with the *x*-axis displaying the number of proteins associated with each pathway and the *y*-axis showing pathway names. Adjusted *p*-values are represented by the color bar, ranging from red for higher values to blue for lower values.

Furthermore, KEGG (Kyoto Encyclopedia of Genes and Genomes) pathway analysis was conducted to identify relevant pathways within the context of network pharmacology. Figure 7 illustrates the counts and *p*-values, with ‘Pathways in cancer’ and ‘MicroRNAs in cancer’ showing the highest counts, indicating a strong association with cancer-related mechanisms.

### 3.4. Molecular Docking

**Figure 8:**
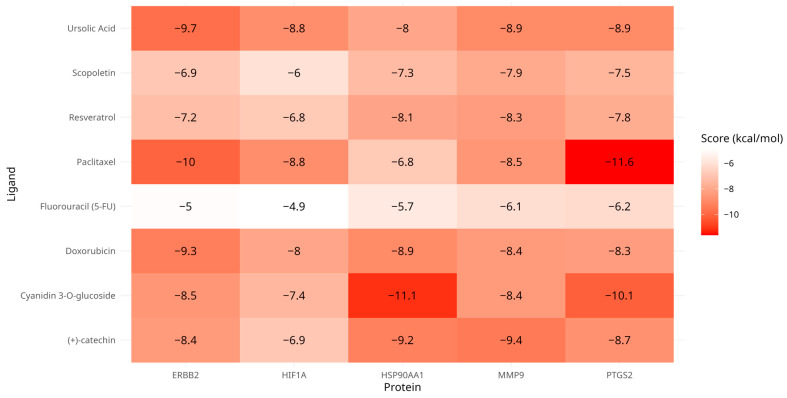
The heatmap displays the molecular docking results of key metabolites from the *Vaccinium* genus with central target proteins. In this representation, darker red shades signify stronger binding affinities between the ligand and receptor, while lighter colors denote weaker binding affinities.

**Figure 9:**
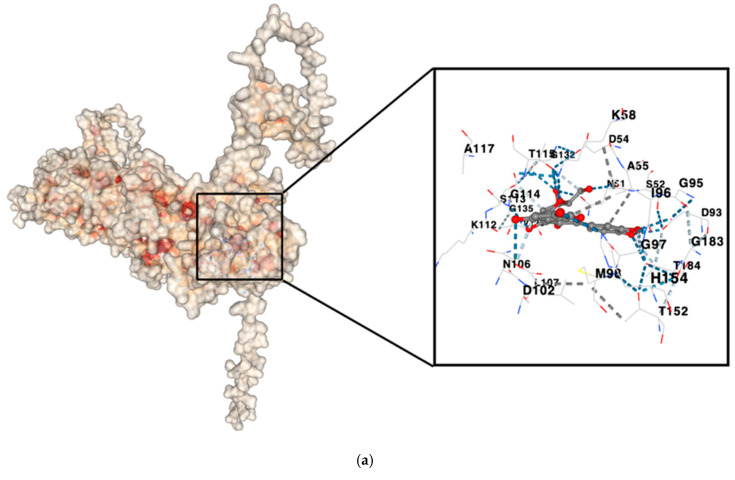

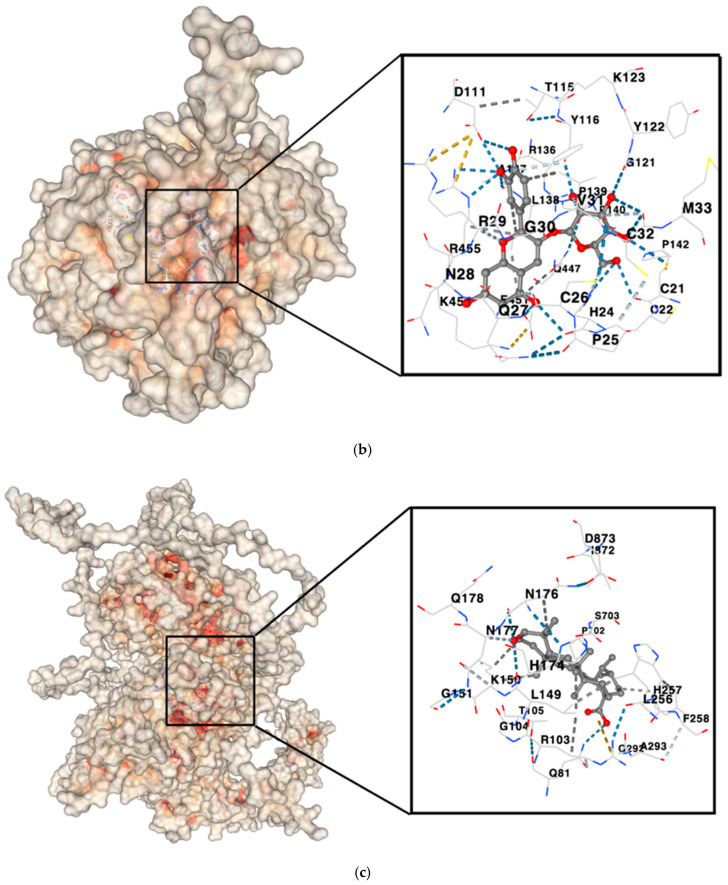
The top 3 ligand–receptor complexes: (**a**) HSP90AA1–cyanidin 3-O-glucoside complex (−11.1 kcal/mol); (**b**) PTGS2–cyanidin 3-O-glucoside complex (−10.1 kcal/mol); and (**c**) ERBB2–ursolic acid complex (−9.7 kcal/mol).

The target proteins implicated in gastric cancer, specifically HIF1A, HSP90AA1, MMP9, ERBB2, and PTGS2, were matched with the key metabolites from the *Vaccinium* genus, including cyanidin 3-O-glucoside, ursolic acid, resveratrol, scopoletin, and (+)-catechin. This evaluation aimed to determine their binding potential to the protein targets predicted by SuperPred. The binding energy values, which reflect the interaction strength between the proteins and the ligands, varied between −6 and −11.1 kcal/mol (see Figure 8). Most metabolites displayed a strong likelihood of stable binding to their respective target proteins. Most of the metabolites exhibited notable binding strengths with their target proteins. Specifically, the complexes HSP90AA1–cyanidin 3-O-glucoside, PTGS2–cyanidin 3-O-glucoside, and PTGS2–ursolic acid demonstrated significant binding affinities, as illustrated in Table 3. Additionally, well-established cancer therapy drugs, including doxorubicin, paclitaxel, and fluorouracil, were docked to the hub proteins. This allowed for a comparative analysis of the binding interactions between *Vaccinium* metabolites and these widely used anticancer agents, specifically in the context of gastric cancer. To further showcase these results, the three most stable receptor–ligand complexes, chosen based on their high affinity, are displayed in Figure 9.

**Figure 10:**
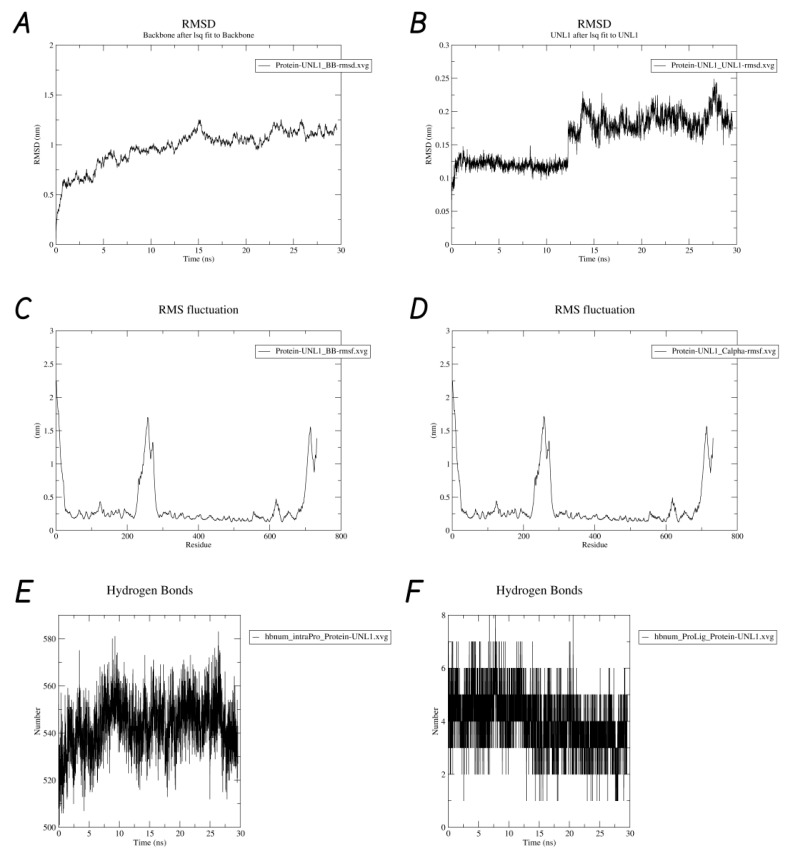

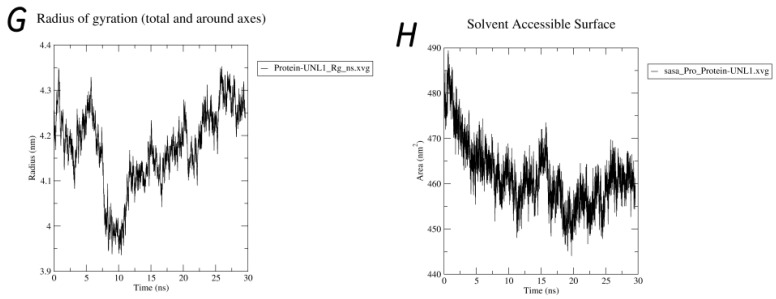
Molecular dynamics simulation results of the HSP90AA1–cyanidin 3-O-glucoside complex. (**A**) RMSD plot of the protein backbone, (**B**) RMSD plot of the ligand, (**C**) RMSF plot of the protein backbone, (**D**) RMSF plot of the Cα atoms, (**E**) hydrogen bond analysis showing the RMSD of intraprotein hydrogen bonds, (**F**) RMSD plot of hydrogen bonds between HSP90AA1 and cyanidin 3-O-glucoside, (**G**) radius of gyration over time, and (**H**) solvent-accessible surface area (SASA) over time.

**Figure 11:**
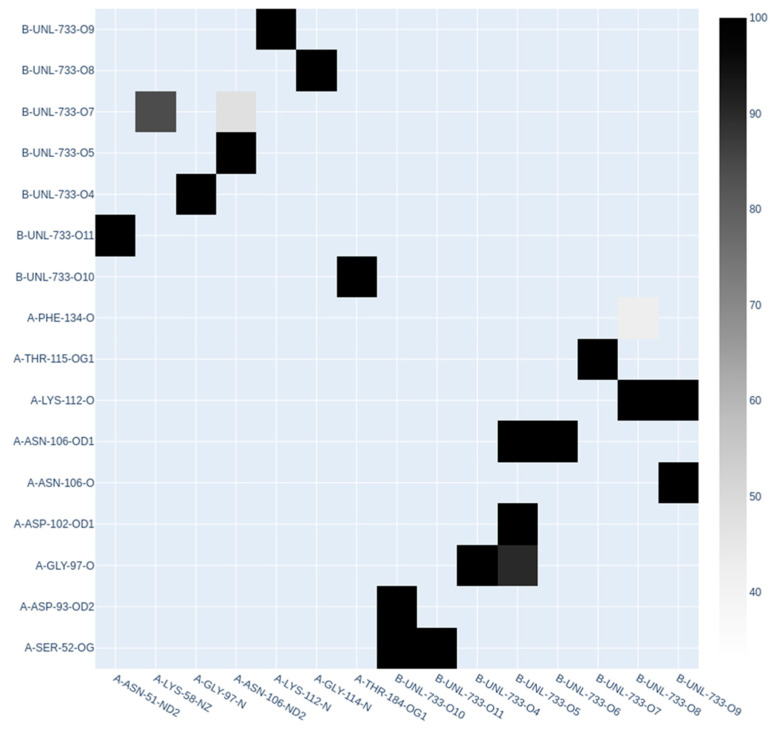
Hydrogen bond matrix of the protein–ligand complex, illustrating the presence and percent occurrence of hydrogen bonds throughout the simulation.

Molecular dynamics simulation of the protein–ligand complex with the highest binding efficiency, HSP90AA1–cyanidin 3-O-glucoside, was performed for a simulation time of 30 ns. Figure 10 illustrates the various results obtained from the simulation. As part of the post-processing analysis, the structural deviations of the HSP90AA1 backbone were investigated to serve as a potential indicator of the complex’s stability. Interestingly, an average RMSD value of 0.972 nm was obtained, with a standard deviation of 0.184 nm. Additionally, the RMSD value for the ligand was found to be 0.158 nm, on average, with a standard deviation of 0.036 nm. The low backbone RMSD value indicates structural stability, while the ligand RMSD suggests a well-retained binding mode throughout the simulation. Similarly, the average RMSF value for the backbone was 0.359 nm, with a standard deviation of 0.361 nm, highlighting the dynamic flexibility of specific regions. The hydrogen bonds within the protein and those in the protein–ligand complex were also analyzed. On average, 543 intraprotein hydrogen bonds were observed during the simulation, while the protein–ligand complex had an average of 4 hydrogen bonds. Figure 11 presents a hydrogen bond matrix analysis, which revealed several key residues consistently involved in interactions during both docking and molecular dynamics simulations. These residues include PHE134, THR115, LYS112, ASN106, ASP102, GLY97, ASP93, SER52, ASN51, LYS58, GLY114, and THR184, emphasizing their potential role in maintaining stable binding and interactions within the system. The average radius of gyration was found to be 4.17 nm, indicating a well-folded protein structure. Additionally, the average solvent-accessible surface area (SASA) was 462 nm^2^, with a standard deviation of 7.14 nm^2^, suggesting only minor conformational changes in the complex over time.

## 4. Discussion

Gastric cancer, like many cancers, is a systemic disease impacting the entire body rather than a single organ. Advances in technology, particularly in computational methods and plant-based compounds or metabolites, have progressively expanded therapeutic options. Currently, integrating RNA sequencing to identify upregulated genes in gastric cancer, along with network pharmacology and plant-based metabolite studies, highlights an innovative, multi-targeted approach to cancer therapy. These methods underscore the evolving diversity of cancer treatment strategies, leveraging computational insights to deepen our understanding of plant-derived compounds’ potential against cancer.

This study integrates RNA sequencing data from gastric cancer with target predictions of *Vaccinium* metabolites using SuperPred. Common proteins between the datasets, including key hub proteins (see Figure 5), offer insights into potential therapeutic targets, highlighting the role of *Vaccinium* metabolites in modulating cancer-related pathways. By predicting targets and identifying upregulated genes in gastric cancer, compound–protein interaction (CPI) networks were constructed, which is also seen in Figure 5. These networks illustrate how selected metabolites interact with shared therapeutic pathways, emphasizing their relevance in the progression from gastritis through atrophy, intestinal metaplasia, dysplasia, and ultimately, gastric cancer. The hierarchical clustering of the compound–protein interaction (CPI) networks in Figure 4 reveals a significant alignment with Correa’s cascade, the histological progression model from chronic gastritis to gastric adenocarcinoma [40]. This analysis identified two distinct clusters: Cluster 1 represents tumor progression, while Cluster 2 corresponds to early-stage gastritis. Correa’s cascade outlines the transition from chronic mucosal inflammation—often induced by *Helicobacter pylori* infection or environmental factors—to atrophic gastritis [38], followed by intestinal metaplasia. In this stage, the gastric epithelium is replaced by goblet cell-rich, intestinal-type epithelium, leading to decreased gastric acidity and pepsinogen I production. Dysplasia emerges next, marked by neoplastic cells with hyperchromatic nuclei, though they remain confined within the lamina propria. The progression culminates in invasive carcinoma, wherein dysplastic cells penetrate the lamina propria, resulting in gastric cancer [38,39].

The RNA sequencing data indicate that Cluster 2 aligns with the early stages of gastric malignancies, suggesting that *Vaccinium* metabolites may influence initial disease development. In contrast, Cluster 1 captures later stages, including dysplasia and adenocarcinoma, indicating potential intervention points in cancer progression. Gene enrichment analysis further supports these findings, with Cluster 1 being enriched in cancer-related terms and Cluster 2 being enriched in immune system-related terms. This study specifically focused on Cluster 1, representing the later stages of gastric carcinoma. This enrichment suggests that *Vaccinium* metabolites may not only affect cancer cell behavior but also modulate the tumor microenvironment, presenting a multifaceted approach to gastric cancer therapy.

The RNA sequencing data highlight several key proteins—HIF1A, HSP90AA1, PTGS2, MMP9, and ERBB2—which are associated with various stages of gastric cancer progression and align with established biomarkers and pathways. HIF1A is consistently linked to primary tumors and early gastric cancer, where high expression correlates with tumor progression, poorly differentiated cell types, and elevated VEGF expression, which all contribute to tumor growth, angiogenesis, and metastasis [41]. Similarly, HSP90AA1 is highly expressed in both primary and early gastric cancer stages, consistent with its role in advanced tumor stages and stress resistance [42]. PTGS2 (COX2) is notably involved in intestinal metaplasia, extensive atrophy, and early gastric cancer, aligning with its established role in promoting tumor progression and inflammation in advanced colorectal cancer [41]. MMP9 is upregulated across multiple stages, including primary tumors, intestinal metaplasia, gastritis, and atrophy, with its expression linked to tumor invasion and metastasis in advanced gastric cancer [43]. Lastly, ERBB2, encoding the HER2 protein, is primarily associated with primary tumors. HER2 overexpression is known to play a significant role in tumor aggressiveness and is linked to poor prognosis in advanced stages of gastric cancer [44].

Moreover, Figure 5a shows that the identified hub proteins from Cluster 1—MMP9, PTGS2, HIF1A, ERBB2, and HSP90AA1—play crucial roles in cancer progression and may serve as effective targets for *Vaccinium* metabolites. It is important to note that these hub proteins are all critically involved in various cancer-related processes and are integral components of oncogenic pathways that regulate processes such as cell proliferation, survival, migration, angiogenesis, and metastasis, which are central to cancer development and progression. MMP9, while known to enhance cancer progression, can paradoxically inhibit growth and metastasis by cleaving signaling molecules, resulting in fragments that act as antagonists and block receptor activation [45]. Additionally, hypoxia-inducible factor 1-alpha (HIF1A) promotes cancer progression through mechanisms such as angiogenesis, metastasis, and alterations in cell proliferation and metabolism [46]. Incorporating these proteins into future studies could inform the development of targeted therapies that leverage the bioactive properties of *Vaccinium* metabolites.

The gene ontology results (see Figure 6) reveal that protein phosphorylation and positive regulation of cell migration exhibit the highest fold enrichment values in biological processes, wherein a high fold enrichment score indicates that the proteins are significantly overrepresented in the context of the disease [47]. The high enrichment in protein phosphorylation indicates its critical role in gastric cancer progression, as it regulates protein activity and signal transduction. Any imbalance in this process can alter protein function, affecting cell proliferation, survival, and migration [48]. Increased phosphorylation levels are associated with aberrant signaling pathways that promote tumor initiation and progression [49]. Similarly, the positive regulation of cell migration underscores its significance in enhancing tumor invasiveness, allowing cancer cells to invade surrounding tissues and spread to distant sites [50]. This suggests that *Vaccinium* metabolites may influence pathways regulating protein activity and enhance cell migration, thereby impacting cancer progression. An analysis of cellular components shows higher enrichment for the cell surface and extracellular exosomes. The significant enrichment of cell surface proteins suggests that *Vaccinium* metabolites modulate cellular interactions and signaling pathways that influence tumor behavior, as they play crucial roles in tumor cell interactions with the microenvironment [51]. For instance, CD44 facilitates vital cell–cell and cell–matrix interactions critical for tumor invasion [52]. Moreover, the enrichment of extracellular exosomes indicates their role in intercellular communication within the tumor microenvironment, promoting tumor growth and invasion while influencing processes such as angiogenesis and therapeutic resistance [53]. In contrast, other components like the nucleoplasm, cytosol, and cytoplasm show moderate enrichment. The molecular functions indicate high enrichment of protein serine kinase activity and related functions, highlighting the importance of phosphorylation-driven signaling pathways in the effects of *Vaccinium* metabolites on gastric cancer progression. These pathways, including the PI3K/AKT/mTOR signaling cascade, are central to regulating cell growth and survival, suggesting potential targets for therapeutic intervention [54].

In the KEGG pathways enrichment analysis (see Figure 7), the pathway in cancer shows the greatest count of common proteins associated with both gastric cancer and *Vaccinium* metabolites. This suggests a strong relevance of these common proteins to cancer pathways, indicating that *Vaccinium* metabolites may play a crucial role in modulating these pathways.

The network pharmacology analysis identified cyanidin-3-O-glucoside (C3G) and ursolic acid as key compounds in *Vaccinium* metabolites with potential therapeutic benefits for gastric cancer. Cyanidin-3-O-glucoside (C3G), the predominant anthocyanin in berries, exhibits antioxidant, anti-inflammatory, and anticancer properties [55,56]. Resveratrol, while abundant in grapes and wine, is also found in blueberries and cranberries [57]. Ursolic acid, common in apple peel and rosemary, is present in lower amounts in cranberries and blackberries [58]. Scopoletin, mainly from noni fruit, occurs in trace levels in elderberries and blackberries [59]. (+)-Catechin, widely distributed in apples, grape seeds, and cocoa, is also notable in blueberries and strawberries [60]. Compared to these sources, berries uniquely combine these compounds, reinforcing their therapeutic potential. In the context of gastric cancer, it promotes apoptosis through reactive oxygen species (ROS)-dependent signaling pathways, such as MAPK, STAT3, and NF-κB. Additionally, it arrests the cell cycle at the G2/M phase via the AKT pathway and prevents cell migration through the β-catenin signaling pathway in human gastric cancer MKN-45 cells [61]. Recent studies have also shown that cyanidin-3-O-glucoside (C3G) induces dose-dependent cytotoxicity in the MCF-7 breast cancer cell line. Flow cytometry revealed over 51.5% apoptosis after 24 h of C3G exposure. Additionally, C3G upregulated apoptosis-related genes like p53, Bax, Caspase-3, CYP1, and CYP2, while downregulating Bcl2. Western blot analysis confirmed a two-fold increase in CYP1 protein levels. These findings suggest that C3G induces apoptosis in breast cancer cells, indicating its potential as a promising cancer therapy candidate [62]. C3G also alleviates symptoms induced by *Helicobacter pylori lipopolysaccharide* (HP LPS) in human gastric epithelial cells by lowering DNA synthesis, promoting apoptosis, and reducing inflammation. On a molecular level, C3G downregulates the expression of toll-like receptors TLR2 and TLR4, which inhibits downstream NF-κB signaling [63]. Furthermore, C3G facilitates cellular senescence by increasing the expression of senescence-associated β-galactosidase (SA-β-gal) and crucial cell cycle regulators like P16, P21, and P53, pushing cancer cells into a senescent state and inhibiting tumor growth [64]. Collectively, these multifaceted mechanisms highlight C3G’s potential as a therapeutic agent in gastric cancer treatment, particularly given the results of this study that C3G interacts with key molecular targets such as PTGS2 and HSP90AA1, which are crucial for cancer cell proliferation and survival [65,66].

Molecular docking analysis revealed binding energies of C3G to PTGS2 (−10.1 kcal/mol) and HSP90AA1 (−11.1 kcal/mol), as well as ursolic acid’s binding to ERBB2 (−9.7 kcal/mol) (see Table 3 and Figure 9). Based on the RNA sequencing data, PTGS2 (cyclooxygenase-2 or COX-2) was identified as an upregulated gene in gastric cancer. PTGS2, an enzyme frequently overexpressed in various cancers [67], plays a crucial role in the synthesis of prostaglandins and thromboxanes, regulating processes key to cancer progression such as apoptotic resistance, increased cell proliferation, angiogenesis, inflammation, and the invasion and metastasis of cancer cells, significantly contributing to tumor growth and development [68,69]. The overexpression of COX-2 is associated with an increased rate of cancer recurrence, reduced survival, and the mediation of tumor cell resistance to treatment, ultimately promoting carcinogenesis [70,71,72]. This phenomenon is closely linked to heightened EGFR activity in tumor cells; specifically, activated EGFR signaling can enhance COX-2 transcription and upregulate the production of prostaglandin E2, further amplifying EGFR activity [73,74]. Given that COX-2 inhibitors have demonstrated antitumor activity across various cancers, the binding of C3G to PTGS2 suggests that it may modulate cancer-promoting pathways, reducing inflammation and inhibiting cancer cell survival by disrupting the inflammatory and proliferative signals mediated by PTGS2. Consequently, C3G emerges as a promising compound in the therapeutic strategy for gastric cancer. Furthermore, the potential of C3G as a therapeutic agent is underscored by its ability to synergistically enhance the efficacy of existing cancer treatments. Recent studies indicate that combining COX-2 inhibitors with conventional treatments can lead to improved clinical outcomes in gastric cancer patients [75,76]. Further investigation into the specific molecular interactions and downstream effects of C3G on COX-2 activity could provide valuable insights into its potential role in integrated cancer therapies.

On the other hand, Heat Shock Protein 90 Alpha Family Class A (HSP90AA1) was also identified from the RNA sequencing data as a crucial component in the pathways associated with gastric cancer. As a member of the Heat Shock Protein 90 (HSP90) family, HSP90AA1 plays a significant role in cellular processes relevant to tumor progression. HSP90 is a molecular chaperone in eukaryotic cells that plays a crucial role in preserving cellular homeostasis [77], including the regulation of receptor tyrosine kinases, metabolic enzymes, and epigenetic regulators essential for the proliferation and survival of cancer cells [78]. It also regulates various cellular processes, including protein folding, intracellular transport, signal transduction, and protein degradation [79]. The overexpression of HSP90AA1 is associated with multiple cancer types [80,81], including gastric cancer. Increased levels of HSP90α are linked to poor prognosis in various cancers and have been found to correlate with higher numbers of CD8+ T cells. Moreover, HSP90AA1 shows significant associations with immune checkpoint genes in pan-cancer analyses [81]. In the context of gastric cancer, elevated HSP90 expression contributes to tumor aggressiveness, highlighting its potential as a therapeutic target. Inhibiting HSP90 could decrease angiogenesis and gastric cancer cell proliferation and help overcome chemotherapy resistance [82]. Therefore, targeting HSP90AA1 with specific inhibitors in gastric cancer treatment represents a promising therapeutic approach. Based on the molecular docking results, C3G interacts with HSP90AA1, demonstrating a binding energy of −11.1, the highest observed in this study (see Figure 9). This result underscores C3G’s potential as a therapeutic agent. By binding to HSP90AA1, C3G may disrupt the chaperone’s function, leading to the destabilization of critical oncoproteins and ultimately inhibiting cancer cell survival and proliferation. Consequently, C3G could serve as a valuable inhibitor in gastric cancer treatment, potentially enhancing the efficacy of existing therapies and improving patient outcomes.

Ursolic acid, another bioactive metabolite from the *Vaccinium* genus identified in this study, exhibits notable anticancer properties. Molecular docking results demonstrated that ursolic acid binds to ERBB2 (HER2) with a binding energy of −9.7. ERBB2, also identified as an upregulated gene in gastric cancer (see Table 3), encodes a 185 kDa transmembrane glycoprotein belonging to the epidermal growth factor receptor (EGFR) family [83]. It is a receptor kinase frequently overexpressed in several cancers, including bladder, breast, lung, salivary, and gastric cancers [84,85,86,87,88]. The overexpression of HER2 in human tumor cells is closely linked to increased angiogenesis and vascular endothelial growth factor (VEGF) expression. Inhibiting VEGF pathways has been demonstrated to reduce tumor growth; for instance, the anti-HER2 antibody trastuzumab effectively diminishes tumor cell proliferation and decreases VEGF expression [89]. The HER2/HER3 heterodimer is especially potent in activating key downstream signaling pathways, such as PI3K/AKT and MAPK, which drive cancer cell proliferation and survival. HER2 expression in gastric cancer can be more heterogeneous than in breast cancer, showing variability in expression patterns within and between tumors [90]. The anticancer effects of ursolic acid are attributed to its ability to trigger apoptosis and disrupt cancer cell division by arresting cells in the G0/G1 phase of the cell cycle. Its anti-metastatic potential was demonstrated through cell migration and invasion assays, where ursolic acid significantly inhibited the migration and invasion of SK-MEL-24 cells by targeting the MAPK/ERK signaling pathway [91]. Additionally, ursolic acid (UA) has demonstrated strong anti-breast cancer (BC) effects across various cancer cell lines, including MCF-7 and MDA-MB-231. In these cells, UA induces apoptosis, arrests the cell cycle, and inhibits cancer cell proliferation by downregulating key proteins such as STAT3, EGFR, and cyclin D1 [92]. Furthermore, UA inhibits cyclin D1 through PI3K/AKT and STAT3 signaling pathways [93]. In MCF-7 cells, UA also promotes autophagy and triggers apoptosis through EIF2AK3-mediated mechanisms and MAPK activation [94]. In MDA-MB-231 cells, UA upregulates apoptotic markers like caspase-3, -8, and -9, as well as Bax, while simultaneously downregulating anti-apoptotic proteins Bcl-2 and PARP [95]. While these findings are focused on breast cancer, they suggest that UA’s mechanisms may also be relevant for other types of cancer, including gastric cancer, by potentially modulating similar pathways involved in tumor progression and apoptosis. Therefore, based on its strong binding affinity to ERBB2 demonstrated through molecular docking, ursolic acid is suggested as a novel therapeutic approach for gastric cancer. By targeting ERBB2, ursolic acid may inhibit key signaling pathways involved in tumor growth and metastasis, presenting a promising alternative or complementary strategy for gastric cancer treatment. Its dual action in inhibiting cancer cell proliferation and reducing metastatic potential further enhances its therapeutic value, positioning ursolic acid as a significant candidate for drug development in gastric cancer.

The binding energies of the top three *Vaccinium* metabolites from the molecular docking study were compared to those of well-established cancer therapy drugs—doxorubicin, paclitaxel, and fluorouracil (5-FU)—used as controls to evaluate their potential as therapeutic agents. Doxorubicin is a prominent chemotherapeutic drug that inhibits cellular replication and triggers p53-dependent apoptosis in cancerous tissues, playing a critical role in treating various cancers [96]. Paclitaxel induces G2/M arrest by binding to β-tubulin, a component of microtubules, thereby stabilizing them, preventing depolymerization, and inhibiting cell mitosis, leading to improved survival rates in cancer patients [97]. Fluorouracil (5-FU), a fluoropyrimidine analog developed over 50 years ago, continues to be a cornerstone of cancer treatment, especially in colorectal cancer and cancers of the breast, respiratory, and digestive tracts, often as part of combination chemotherapy regimens [98]. The binding energies of the *Vaccinium* metabolites were compared to these established drugs, it was evident that some of the metabolites exhibited binding energies similar to or even stronger than those of the controls. Cyanidin 3-O-glucoside, for example, demonstrated a binding energy of −11.1 kcal/mol, which closely matches that of paclitaxel (−11.6 kcal/mol) and surpasses doxorubicin (−9.3 kcal/mol) and fluorouracil (−6.2 kcal/mol). Similarly, ursolic acid, with a binding energy of −9.7 kcal/mol, showed significant potential when compared to doxorubicin and fluorouracil. These findings suggest that the *Vaccinium* metabolites could interact with cancer-related targets in a manner similar to clinically approved drugs, potentially serving as viable alternatives or complementary agents in cancer treatment. The close binding energies between these natural compounds and established chemotherapeutics indicate their promise as therapeutic agents. This strengthens the case for further exploration of plant-based compounds in the development of novel cancer therapies, potentially offering a safer and more sustainable approach to treatment with fewer side effects compared to synthetic drugs.

Molecular dynamics simulation is a valuable tool for exploring the dynamic behavior and stability of biomolecular complexes, particularly for processes such as ligand binding, conformational changes, protein folding, and membrane transport [99]. In this study, the HSP90AA1–cyanidin 3-O-glucoside complex was analyzed over 30 ns to assess its structural integrity and binding efficiency. The backbone RMSD averaged 0.972 nm (SD: 0.184 nm), indicating stable protein conformation, while the ligand RMSD was 0.158 nm (SD: 0.036 nm), reflecting a consistently retained binding mode. Backbone flexibility (RMSF: 0.359 nm, SD: 0.361 nm) revealed dynamic regions within the protein. Stable hydrogen bonding networks, including an average of 543 intraprotein and 4 protein–ligand hydrogen bonds, highlighted residues like PHE134, THR115, and ASN106 as key contributors. Structural metrics such as the radius of gyration (4.17 nm) and SASA (462 nm^2^, SD: 7.14 nm^2^) confirmed a well-folded protein–ligand complex with minimal conformational changes [100,101]. These findings underscore the robust stability and effective binding characteristics of the complex, aligning with HSP90AA1’s role as a molecular chaperone that supports protein folding and stabilizes oncogenic proteins [102,103]. The involvement of residues like PHE134 and ASP93 in hydrogen bonding emphasizes their critical roles in maintaining interaction specificity and potentially influencing client protein or co-chaperone binding. Their location in functional domains, such as ATP- and substrate-binding regions, further highlights their importance in HSP90AA1’s chaperone activity [104]. The stable binding of cyanidin 3-O-glucoside, as evidenced by low ligand RMSD and minimal conformational changes, suggests it may inhibit HSP90AA1’s chaperone function by disrupting its conformational dynamics or client protein interactions [105]. This aligns with experimental trends observed for small-molecule inhibitors targeting the N-terminal ATP-binding domain, such as those reported by Whitesell et al. (1994) [105] and Neckers and Trepel (2013) [106]. While direct experimental data for cyanidin 3-O-glucoside are unavailable, its binding specificity and stability parallel findings for known inhibitors that destabilize oncogenic client proteins like HER2, AKT, and HIF-1α [107]. These results provide a foundation for further exploration of cyanidin 3-O-glucoside as a potential therapeutic agent targeting HSP90AA1. Lastly, berries, particularly those from the *Vaccinium* genus, provide a unique combination of bioactive compounds, which have been individually linked to anticancer effects. This is further supported by the fact that the metabolites from the *Vaccinium* genus appear to target pathways upregulated in precancerous lesions of the stomach [108]. The potential synergistic interactions among these metabolites may enhance their therapeutic impact, as observed in previous studies on dietary polyphenols and cancer prevention [60,109,110,111,112].

## 5. Conclusions

This study bridges the gap between traditional plant-based therapies and modern computational methods to uncover the therapeutic potential of *Vaccinium* metabolites in gastric cancer prevention and treatment. By leveraging RNA sequencing, network pharmacology, molecular docking, and molecular dynamics, this study has elucidated how bioactive metabolites like C3G and ursolic acid interact with key cancer-related proteins, such as PTGS2, HSP90AA1, and ERBB2. These findings contribute to the growing body of evidence supporting the role of natural compounds in modulating cancer pathways and highlight the promise of C3G as a potential therapeutic agent, particularly due to its interaction with critical targets in gastric cancer.

Molecular dynamics simulations further validated the binding stability and structural integrity of the HSP90AA1-C3G complex, revealing low RMSD values, consistent hydrogen bonding, and minimal conformational changes. These results indicate that C3G effectively interacts with HSP90AA1 without disrupting its native conformation, underscoring its therapeutic potential in targeting oncogenic pathways.

Although no novel anticancerogenic metabolites were identified in this study, the findings reinforce the potential of *Vaccinium* species as natural therapeutics when incorporated into the diet. The bioactive compounds present in these berries may exert synergistic effects on cancer-related pathways, supporting their role in dietary strategies for cancer prevention. Further research is warranted to explore these combinatory effects and their implications for clinical applications.

This research highlights the promise of integrating plant-based compounds with computational techniques to develop more effective and sustainable cancer therapies. The bioactive properties of *Vaccinium* metabolites, coupled with computational insights, present a promising frontier in cancer therapy.

Although limited to computational methods, this study establishes a foundation for future in vitro and in vivo studies to validate these interactions and overcome practical challenges like bioavailability. Further exploration through experimental validation may ultimately support drug development in gastric cancer using *Vaccinium* metabolites.

## Figures and Tables

**Table 1:** Key bioactive anticancer metabolites from the *Vaccinium* genus and their chemical structures.

No.	Compound	Lipinski’s Rule	Caco-2	2D Chemical Structure
1	Resveratrol	Passed	−4.92	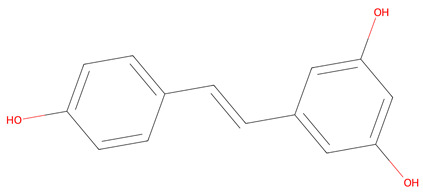
2	Ursolic Acid	Passed	−5.54	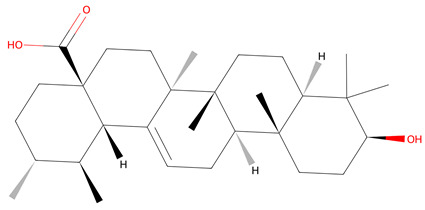
3	Scopoletin	Passed	−4.71	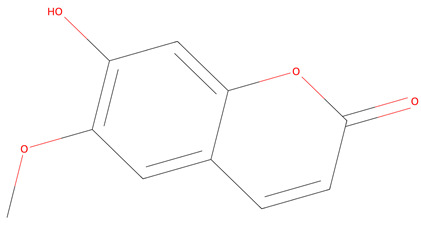
4	(+)-catechin	Passed	−6.17	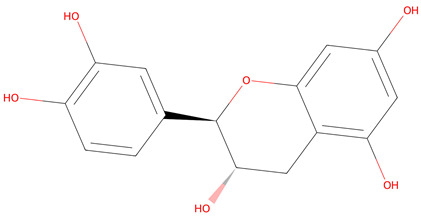
5	Cyanidin 3-O-glucoside *	Failed	−6.35	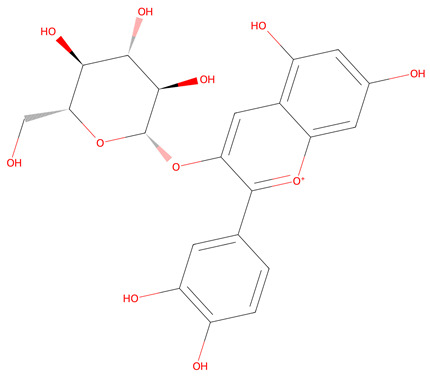

* Conditional acceptance.

**Table 2:** Top hub proteins from the PPI with gene name and cluster membership.

Symbol	Gene Name	Cluster
MMP9	Matrix metallopeptidase 9	1, 2
PTGS2	Prostaglandin-endoperoxide synthase 2	1, 2
HIF1A	Hypoxia-inducible factor 1 subunit alpha	1
ERBB2	Erb-b2 receptor tyrosine kinase 2	1
HSP90AA1	Heat shock protein 90 alpha family class A member 1	1
CXCR4	C-X-C motif chemokine receptor 4	2
ESR1	Signal transducer and activator of transcription 1	2
STAT1	Signal transducer and activator of transcription 1	2

**Table 3:** Binding sites and binding energies of the top 3 receptor–ligand complexes compared to control drugs.

Ligand	Receptor	Binding Energy (kcal/mol)	Binding Sites
Cyanidin 3-O-glucoside	HSP90AA1	−11.1	Chain A: LEU48 ILE49 ASN51 SER52 ASP54 ALA55 LYS58 ILE91 ASP93 GLY95 ILE96 GLY97 MET98 ASP102 ASN106 LEU107 THR109 ALA111 LYS112 SER113 GLY114 THR115 LYS116 ALA117 PHE118 GLY132 GLN133 PHE134 GLY135 VAL136 GLY137 PHE138 TYR139 VAL150 THR152 HIS154 GLY183 THR184 VAL186
Cyanidin 3-O-glucoside	PTGS2	−10.1	Chain A: CYS21 CYS22 HIS24 PRO25 CYS26 GLN27 ASN28 ARG29 GLY30 VAL31 CYS32 MET33 ASP43 CYS44 THR45 ARG46 THR47 GLY48 PHE49 SER107 HIS108 LEU109 ILE110 ASP111 SER112 PRO113 THR115 TYR116 GLY121 TYR122 LYS123 ARG136 ALA137 LEU138 PRO139 PRO140 PRO142 GLN356 GLN358 GLN447 GLU451 LYS454 ARG455 MET457
Ursolic Acid	ERBB2	−9.7	Chain A: GLN81 ARG103 GLY104 THR105 LEU149 LYS150 GLY151 HIS174 ASN176 ASN177 GLN178 CYS255 LEU256 HIS257 PHE258 LEU266 GLY292 ALA293 PRO702 SER703 ARG840 ILE872 ASP873
Paclitaxel *	PTGS2	−11.8	Chain A: ASN19 CYS21 ARG29 VAL31 CYS32 MET33 SER34 ASP43 THR45 ARG46 THR47 ILE110 ASP111 SER112 PRO113 PRO114 THR115 TYR116 ASP119 TYR120 GLY121 TYR122 LYS123 SER124 TRP125 PHE128 ALA137 PRO139 PRO140 VAL141 PRO142 ASP143 ASP144 GLN447 ARG455
Fluorouracil (5-FU) *	PTGS2	−6.2	Chain A: TYR134 PHE184 ALA185 PHE186 ALA188 GLN189 THR192 HIS193 PHE196 LYS197 THR198 HIS200 GLN275 VAL277 ASN368 TYR371 HIS372 TRP373 HIS374 LEU376 LEU377 LYS432 VAL433 GLN435 ALA436 GLN440 LEU597 LYS598 GLU599 ARG600 SER601 THR602 GLU603 LEU604
Doxorubicin *	ERBB2	−9.3	Chain A: LEU726 GLY727 SER728 GLY729 ALA730 PHE731 GLY732 VAL734 ALA751 LYS753 LEU755 ARG756 ASN758 ILE767 GLU770 SER783 THR798 GLN799 LEU800 MET801 GLY804 CYS805 LEU807 ASP808 HIS809 ARG811 ARG844 ASP845 ARG849 ASN850 VAL851 LEU852 THR862 ASP863 LEU866 ARG868 TYR877 ALA879 ASP880 GLY881 GLY882 LYS883 VAL884 PRO885 ILE886 LYS887 TRP888 GLU914 GLY919 ALA920 LYS921 PRO922 PHE1004 PRO1241 THR1242 ALA1243 GLU1244 ASN1245 PRO1246 GLU1247 TYR1248

* Established cancer therapy drugs used as controls for comparison.

## Data Availability

The RNA count data used in this paper are all publicly available in the R library recount3. Any other data and results presented in this study are available upon request from the authors.

## Supplementary Materials

The following supporting information can be downloaded at https://www.mdpi.com/article/10.3390/life15030406/s1: Figure S1: Compound–pathway interaction network of *Vaccinium* metabolites and upregulated genes of gastritis; Figure S2: Compound–pathway interaction network of *Vaccinium* metabolites and upregulated genes of atrophy; Figure S3: Compound–pathway interaction network of *Vaccinium* metabolites and upregulated genes of extensive atrophy; Figure S4: Compound–pathway interaction network of *Vaccinium* metabolites and upregulated genes of intestinal metaplasia; Figure S5: Compound–pathway interaction network of *Vaccinium* metabolites and upregulated genes of low-grade dysplasia; Figure S6: Compound–pathway interaction network of *Vaccinium* metabolites and upregulated genes of high-grade dysplasia; Figure S7: Compound–pathway interaction network of *Vaccinium* metabolites and upregulated genes of early gastric cancer; Figure S8: Compound–pathway interaction network of *Vaccinium* metabolites and upregulated genes of primary gastric cancer tumor; Figure S9: Alluvial representation of the compound–cluster–pathway network; Table S1: Screened *Vaccinium* metabolites.
