# Supplementary material for: Berries as Nature’s Therapeutics: Exploring the Potential of Vaccinium Metabolites in Gastric Cancer Treatment Through Computational Insights
Source: Life (Basel). 2025 Mar 5;15(3):406. doi: 10.3390/life15030406 (PMC11944152; doi:10.3390/life15030406)
Supplement: Supplementary file 1 [file life-15-00406-s001.zip › life-3463065-supplementary.pdf]

Gastritis

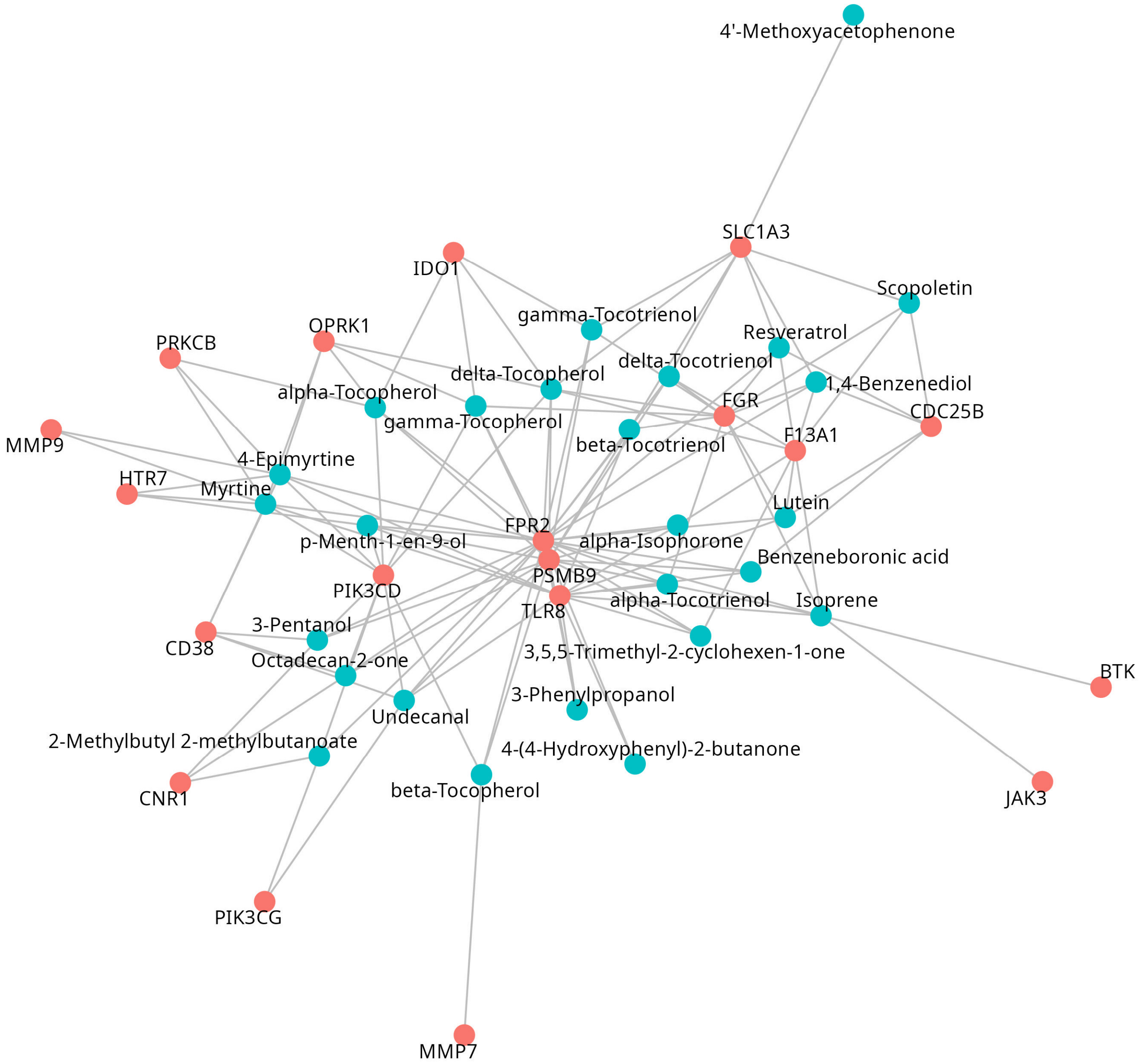

Figure S1. Compound-Pathway Interaction Network of *Vaccinium* metabolites and Upregulated Genes of Gastritis

Atrophy

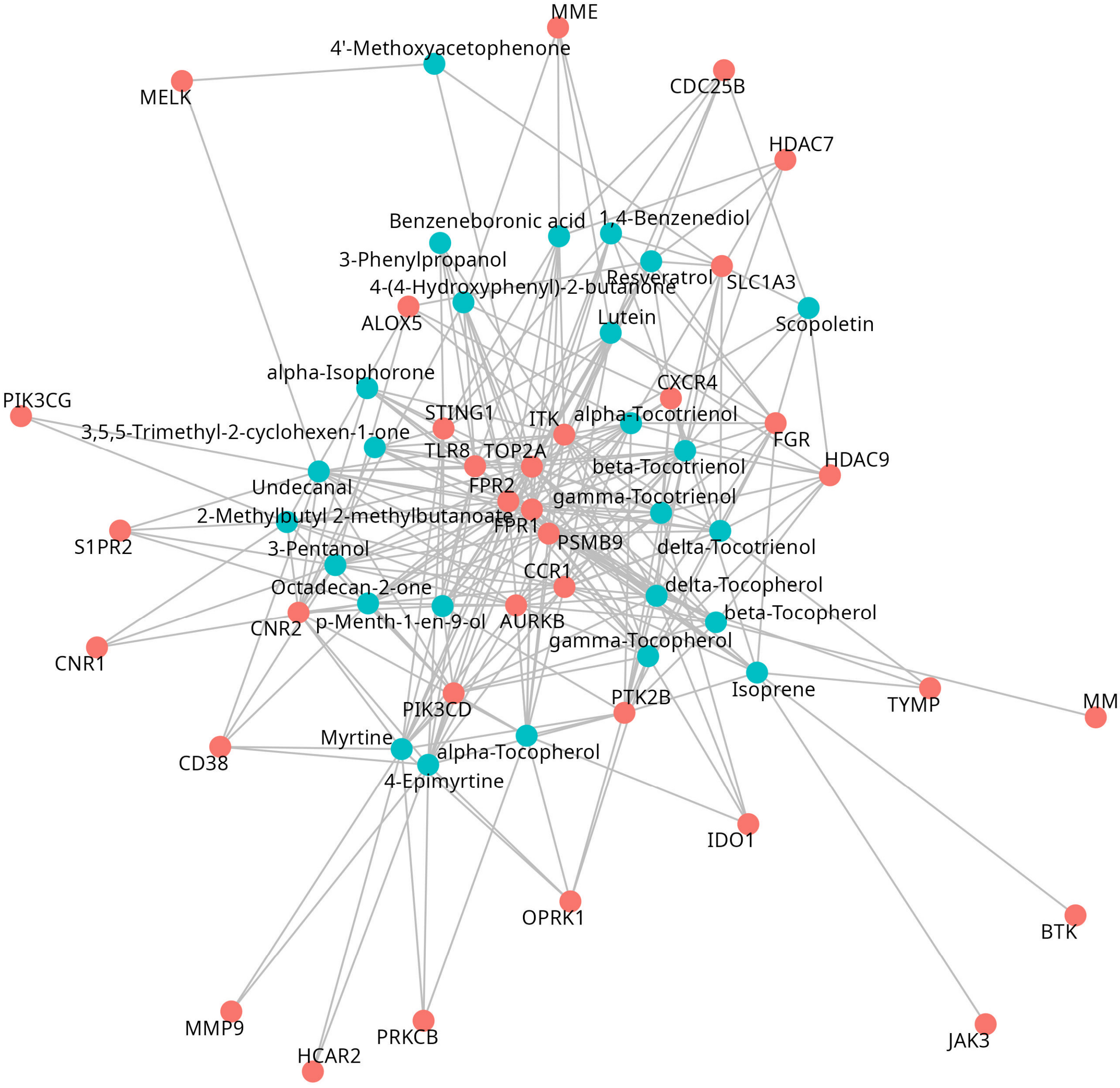

Figure S2. Compound-Pathway Interaction Network of Vaccinium metabolites and Upregulated Genes of Atrophy

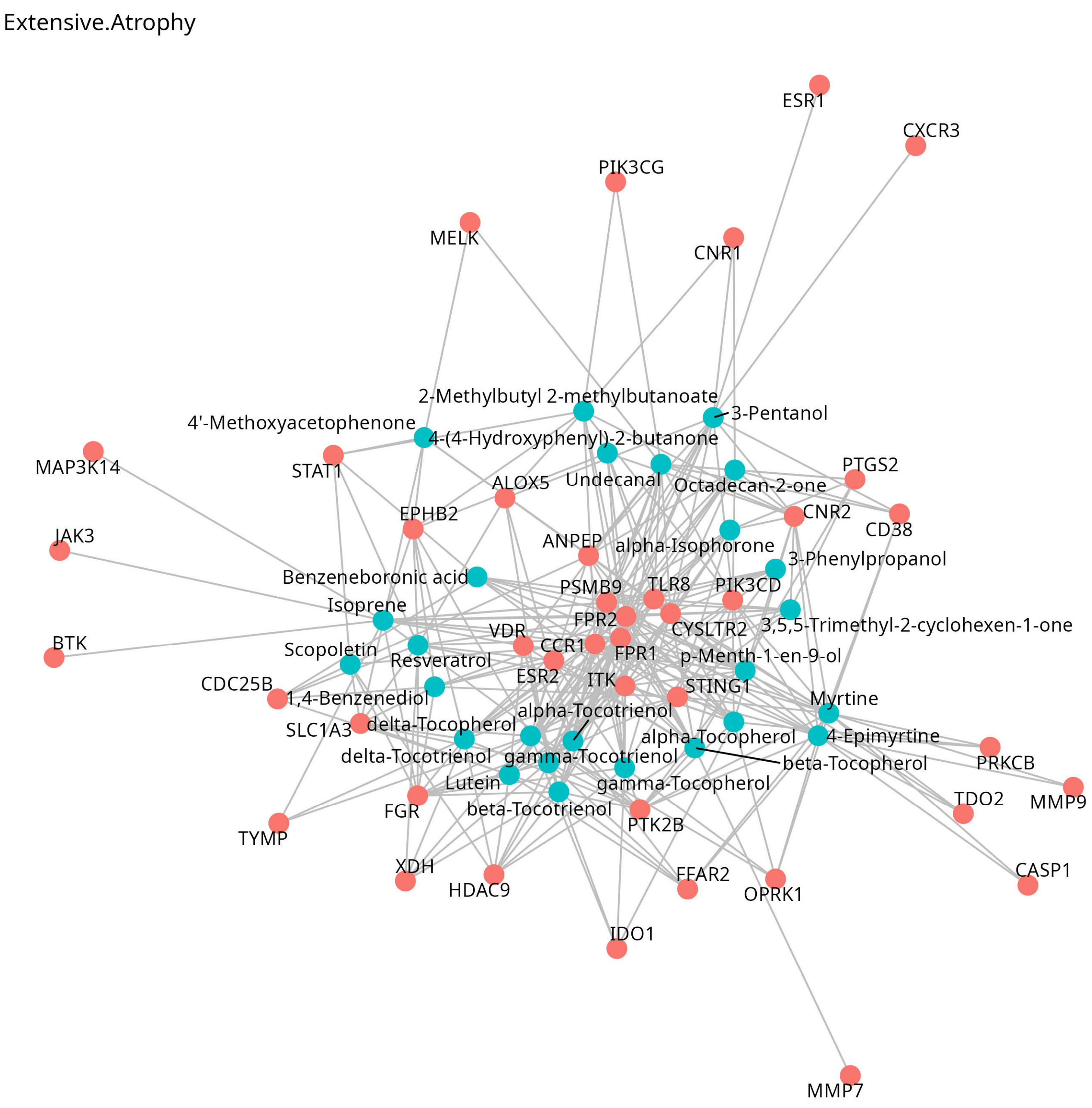

Figure S3. Compound-Pathway Interaction Network of *Vaccinium* metabolites and Upregulated Genes of Extensive Atrophy

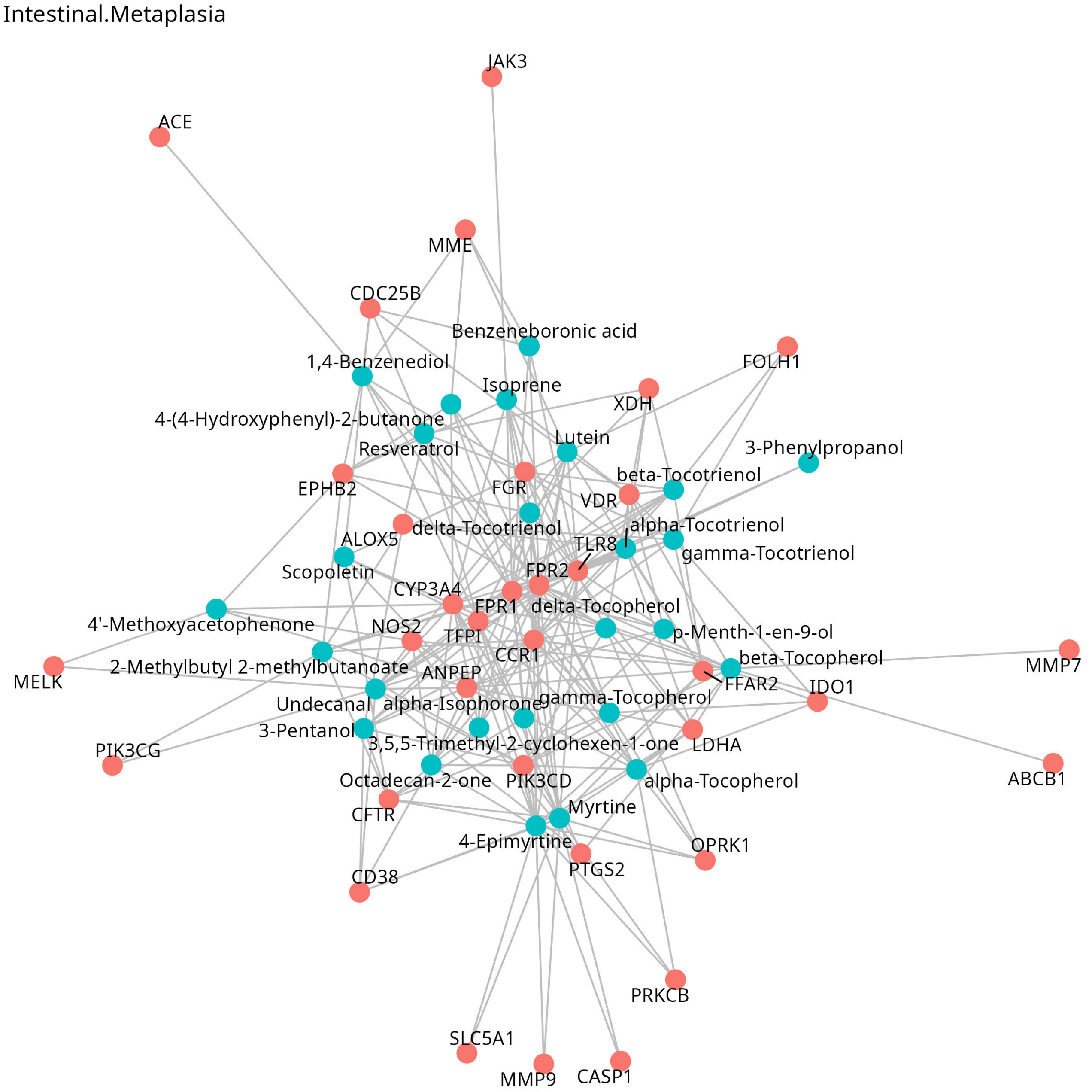

Figure S4. Compound-Pathway Interaction Network of *Vaccinium* metabolites and Upregulated Genes of Intestinal Metaplasia

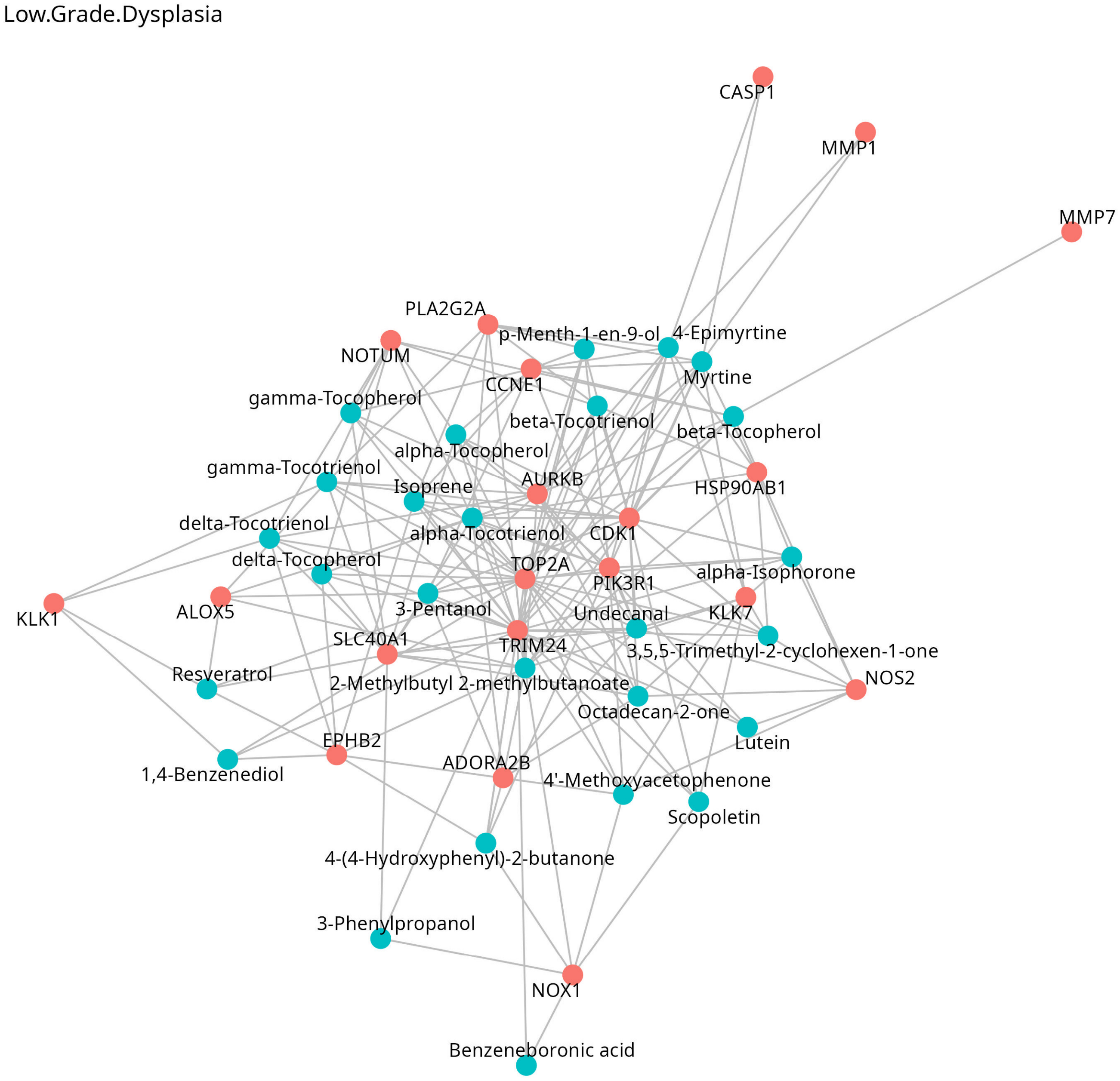

Figure S5. Compound-Pathway Interaction Network of *Vaccinium* metabolites and Upregulated Genes of Low Grade Dysplasia

High.Grade.Dysplasia

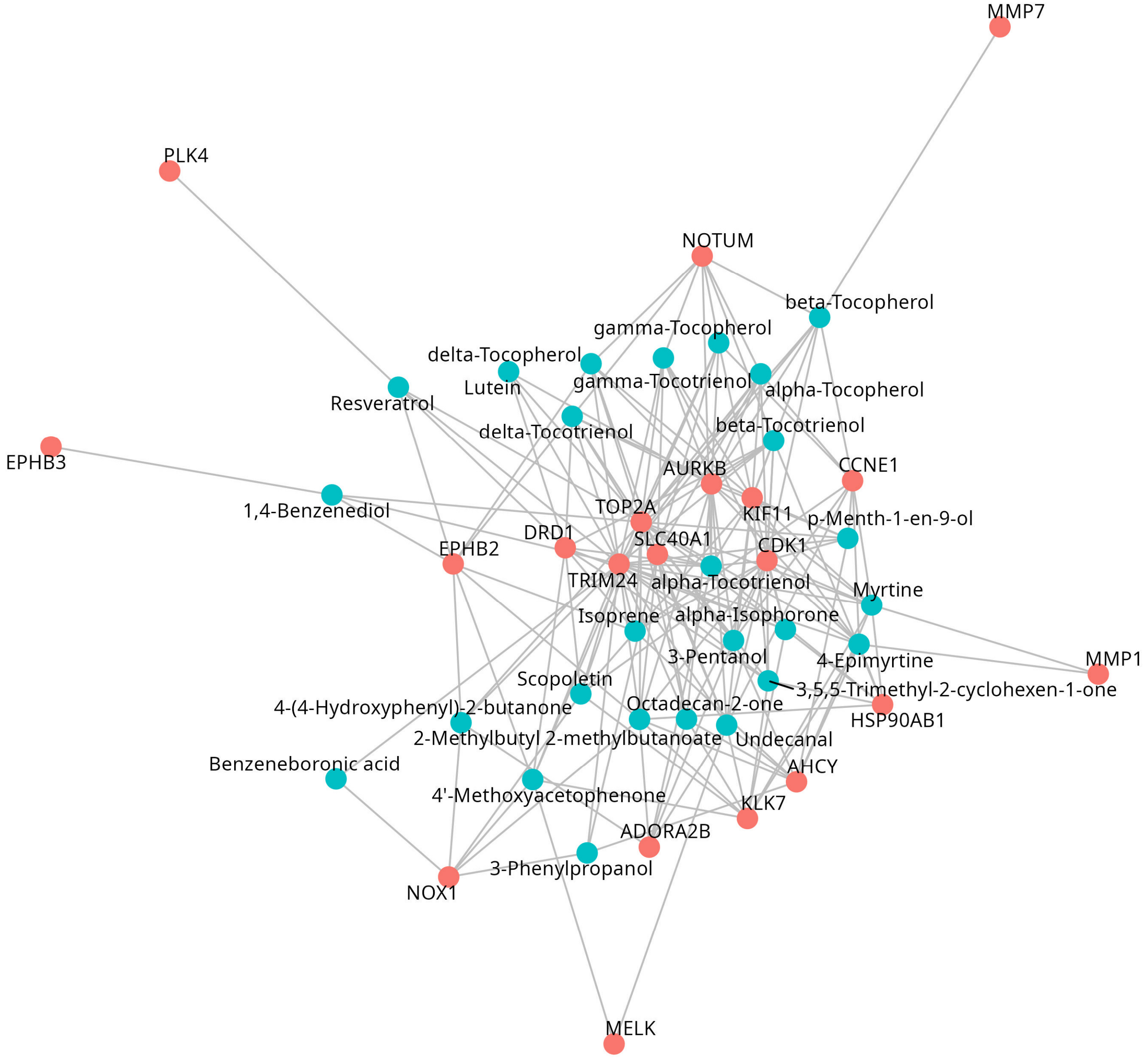

Figure S6. Compound-Pathway Interaction Network of *Vaccinium* metabolites and Upregulated Genes of High Grade Dysplasia

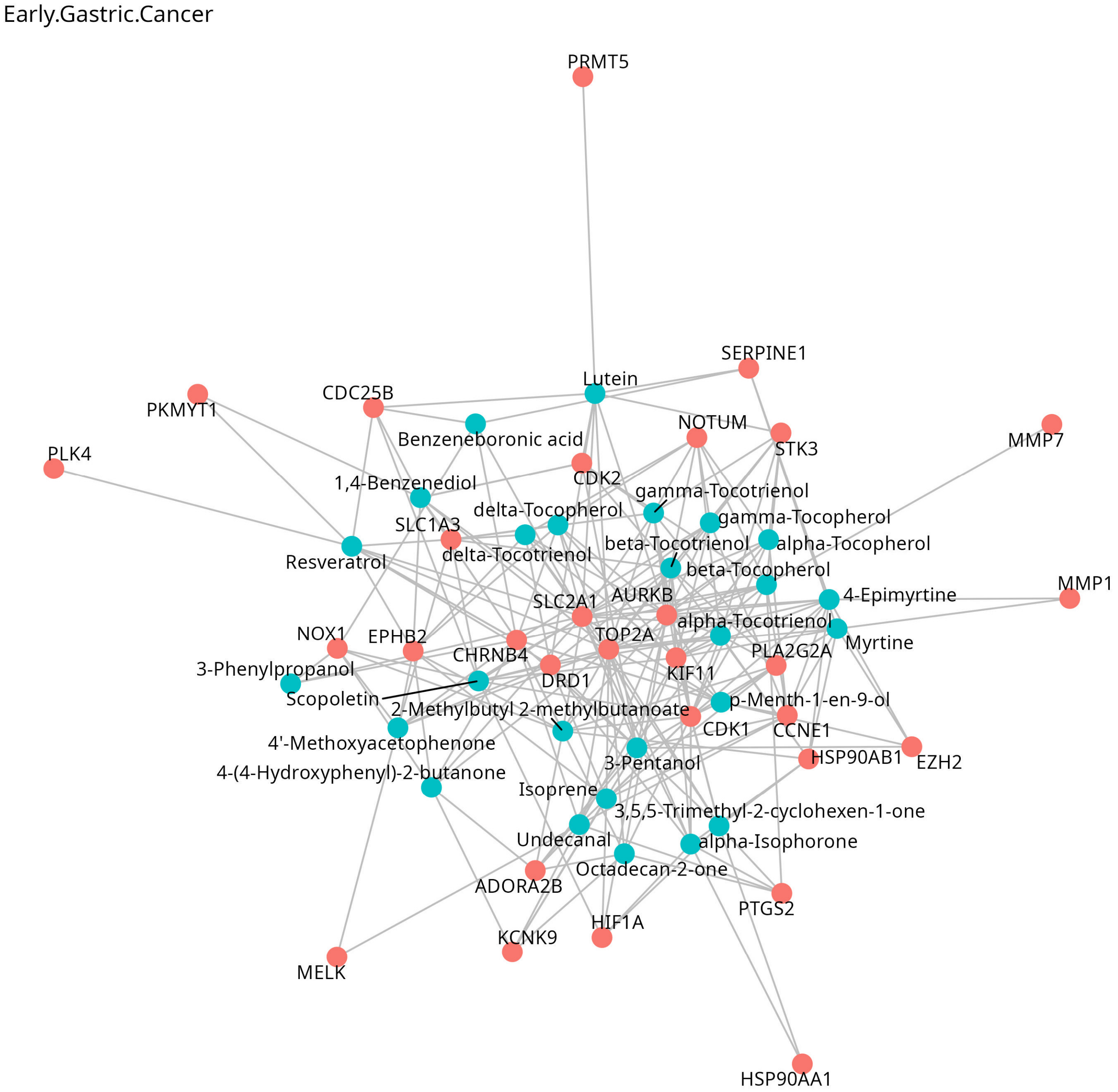

Figure S7. Compound-Pathway Interaction Network of *Vaccinium* metabolites and Upregulated Genes of Early Gastric Cancer

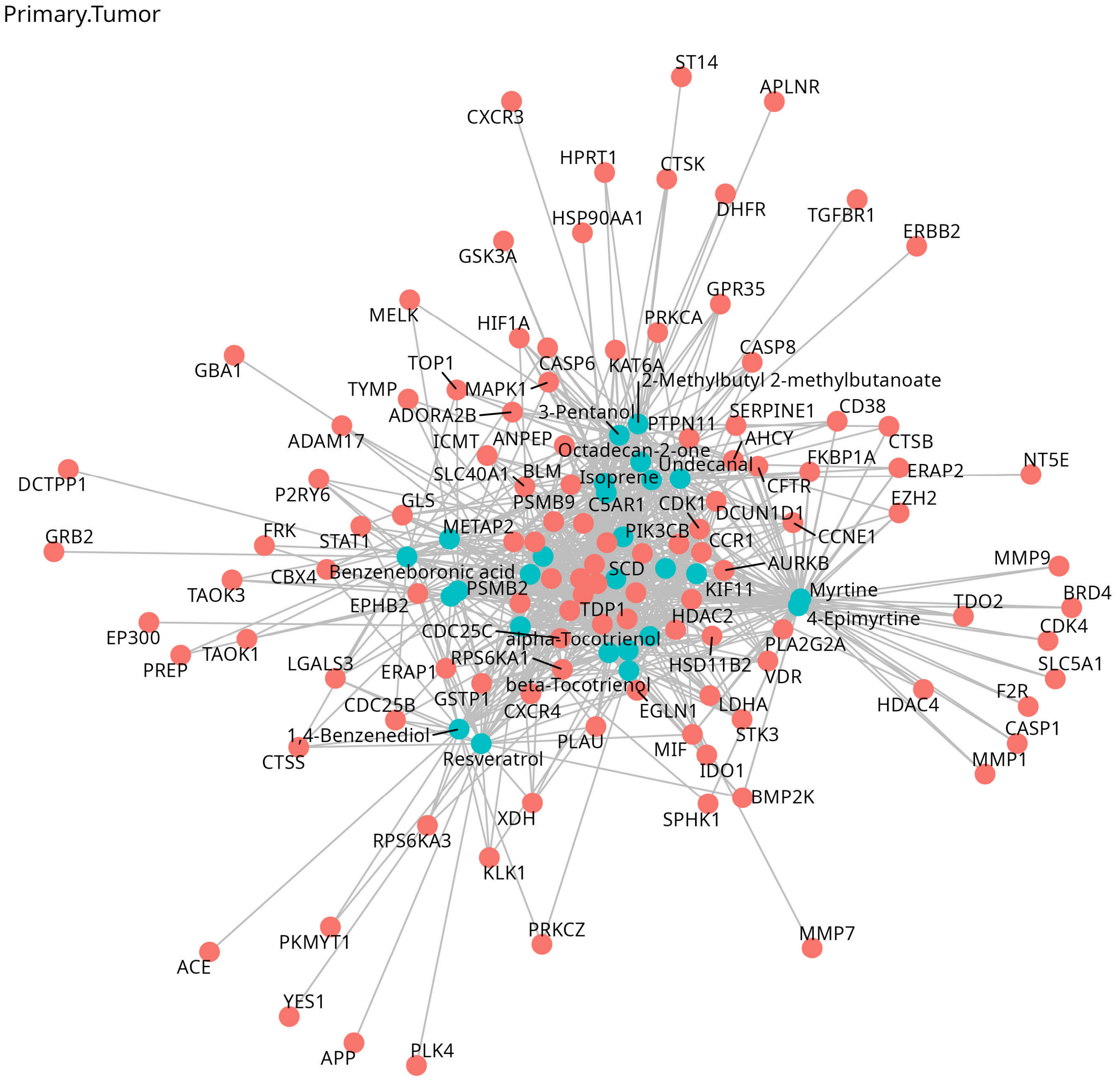

Figure S8. Compound-Pathway Interaction Network of *Vaccinium* metabolites and Upregulated Genes of Primary Gastric Cancer Tumor

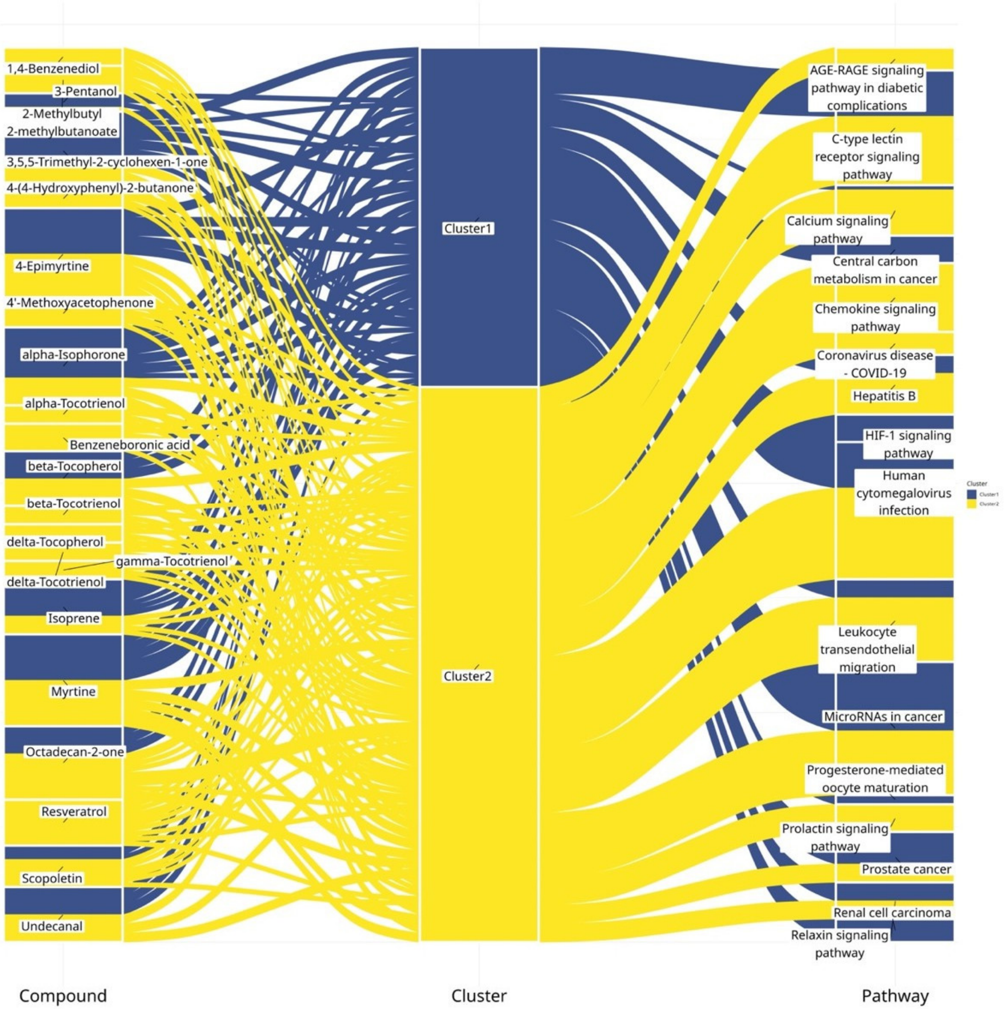

Figure S9. Alluvial Representation of the Compound-Cluster-Pathway Network.

Table S1. Screened *Vaccinium* Metabolites

Organism	Metabolite	MW	TPSA	Lipinski	caco2
Vaccinium vitis-idaea	(+)-catechin	290.08	110.38	Passed	-6.17
Vaccinium vitis-idaea	(-)-Epicatechin	290.08	110.38	Passed	-6.47
Vaccinium myrtillus L.	Cyanidin 3-O-glucoside	449.11	191.6	Failed	-6.35
Vaccinium spp.	Cyanin	611.16	270.75	Failed	-6.69
Vaccinium vitis-idaea	Scopoletin	192.04	59.67	Passed	-4.71
Vaccinium vitis-idaea	Arbutin	272.09	119.61	Passed	-6.13
Vaccinium vitis-idaea	1,4-Benzenediol	110.04	40.46	Passed	-5.08
Vaccinium angustifolium	Resveratrol	228.08	60.69	Passed	-4.92
Vaccinium vitis-idaea	Procyanidin A2	576.13	209.76	Failed	-7.08
Vaccinium myritillus	Procyanidin B4	578.14	220.76	Failed	-7.09
Vaccinium spp.	Monotropein	390.12	186.37	Failed	-6.27
Vaccinium macrocarpon	Ursolic acid	456.36	57.53	Passed	-5.54
Vaccinium macrocarpon	Lutein	568.43	40.46	Passed	-5.08
Vaccinium myritillus	Avicularin	434.08	190.28	Failed	-6.26
Vaccinium myrtillus	Guaijaverin	434.08	190.28	Failed	-6.14
Vaccinium angustifolium	Cyanidin	287.06	112.45	Passed	-5.77
Vaccinium japonicum	Pelargonidin 3-arabinoside	403.1	151.14	Passed	-6.24
Vaccinium spp.	Fragarin	433.11	171.37	Passed	-6.42
Vaccinium spp.	Cyanidin 3-arabinoside	419.1	171.37	Passed	-6.17
Vaccinium spp.	Cyanidin 3-O-beta-D-galactopyranoside	449.11	191.6	Failed	-6.37
Vaccinium padifolium	Cyanidin 3-(2G-xylosylrutinoside)	727.21	309.44	Failed	-6.5
Vaccinium padifolium	Cyanidin 3-sambubioside-5-glucoside	743.2	329.67	Failed	-6.9
Vaccinium macrocarpon	Peonidin 3-arabinoside	433.11	160.37	Passed	-6.17
Vaccinium arboreum	Peonidin-3-galactoside	463.12	180.6	Failed	-6.22
Vaccinium spp.	Oxycoccicyanin	463.12	180.6	Failed	-6.3
Vaccinium padifolium	Peonidin 3-sambubioside	595.17	239.52	Failed	-6.46
Vaccinium angustifolium	Delphinidin 3-arabinoside	435.09	191.6	Failed	-6.24
Vaccinium padifolium	Empetrin	465.1	211.83	Failed	-6.39
Vaccinium spp.	Myrtillin	465.1	211.83	Failed	-6.38
Vaccinium padifolium	Delphinidin 3-rhamnoside	449.11	191.6	Failed	-6.26
Vaccinium padifolium	Delphinidin 3-sambubioside-5-glucoside	759.2	349.9	Failed	-6.85
Vaccinium arboreum	Petunidin 3-arabinoside	449.11	180.6	Failed	-6.16
Vaccinium myrtillus L.	Petunidin 3-O-beta-D-galactopyranoside	479.12	200.83	Failed	-6.24
Vaccinium spp.	Petunidin 3-glucoside	479.12	200.83	Failed	-6.29
Vaccinium padifolium	Malvidin 3-arabinoside	463.12	169.6	Failed	-6.12
Vaccinium corymbosum	Primulin	493.13	189.83	Failed	-6.12
Vaccinium spp.	Oenin	493.13	189.83	Failed	-6.21
Vaccinium padifolium	Malvidin 3-rutinoside	639.19	248.75	Failed	-6.22
Vaccinium macrocarpon Aiton	delta-Tocopherol	402.35	29.46	Passed	-5.01
Vaccinium macrocarpon Aiton	beta-Tocopherol	416.37	29.46	Passed	-5.01
Vaccinium macrocarpon Aiton	gamma-Tocopherol	416.37	29.46	Passed	-4.97
Vaccinium macrocarpon Aiton	alpha-Tocopherol	430.38	29.46	Passed	-5.02

Vaccinium oxycoccus	Leptosin	462.12	175.37	Failed	-6.15
Vaccinium vitis-idaea	(+)-Gallocatechin	306.07	130.61	Passed	-6.44
Vaccinium vitis-idaea	(-)-Epigallocatechin	306.07	130.61	Passed	-6.66
Vaccinium vitis-idaea	Procyanidin B6	578.14	220.76	Failed	-7.04
Vaccinium vitis-idaea	Procyanidin B3	578.14	220.76	Failed	-6.87
Vaccinium vitis-idaea	Procyanidin B8	578.14	220.76	Failed	-6.94
Vaccinium vitis-idaea	Procyanidin B7	578.14	220.76	Failed	-7.23
Vaccinium vitis-idaea	Procyanidin B1	578.14	220.76	Failed	-7.1
Vaccinium vitis-idaea	Procyanidin B5	578.14	220.76	Failed	-7.14
Vaccinium myritillus	Procyanidin B2	578.14	220.76	Failed	-7.22
Vaccinium vitis-idaea	Proanthocyanidin A1	576.13	209.76	Failed	-6.92
Vaccinium vitis-idaea	Cinnamtannin D1	864.19	320.14	Failed	-7.7
Vaccinium vitis-idaea	Cinnamtannin B1	864.19	320.14	Failed	-7.8
Vaccinium vitis-idaea	Epicatechin-(4beta->6)-epicatechin-(2beta->7,4beta->8)-catechin	864.19	320.14	Failed	-7.78
Vaccinium vitis-idaea	Epicatechin-(4beta->8)-epicatechin-(2beta->7,4beta->8)-catechin	864.19	320.14	Failed	-7.69
Vaccinium vitis-idaea	Epicatechin-(2beta->7,4beta->8)-epicatechin-(4beta->8)-epicatechin-(4beta->6)-catechin	1152.25	430.52	Failed	-8.56
Vaccinium vitis-idaea	Epicatechin-(2beta->7,4beta->8)-[epicatechin-(4beta->8)]2-catechin	1152.25	430.52	Failed	-8.44
Vaccinium vitis-idaea	Cinnamtannin D2	1152.25	430.52	Failed	-8.44
Vaccinium vitis-idaea	Cinnamtannin B2	1152.25	430.52	Failed	-8.53
Vaccinium bracteatum	Vaccinoside	536.15	212.67	Failed	-6.2
Vaccinium myrtillus	p-Menth-1-en-9-ol	154.14	20.23	Passed	-4.82
Vaccinium padifolium	Petunidin 3-sambubioside	611.16	259.75	Failed	-6.45
Vaccinium padifolium	Malvidin 3-O-beta-D-sambubioside	625.18	248.75	Failed	-6.31
Vaccinium padifolium	Peonidin 3-[6-(rhamnosyl)-2-(xylosyl)glucoside]	741.22	298.44	Failed	-6.52
Vaccinium padifolium	Petunidin 3-[6-(rhamnosyl)-2-(xylosyl)glucoside]	757.22	318.67	Failed	-6.51
Vaccinium myrtillus L.	4-Epimyrtine	167.13	20.31	Passed	-4.58
Vaccinium arctostaphylos	3,5-Dicaffeoylquinic acid	516.13	211.28	Failed	-6.59
Vaccinium microcarpa	alpha-Isophorone	138.1	17.07	Passed	-4.66
Vaccinium myrtillus	Lyoniside	552.22	176.76	Failed	-6.21
Vaccinium myrtillus L.	Petunidin 3-O-alpha-L-arabinopyranoside	449.11	180.6	Failed	-6.12
Vaccinium vitis-idaea	Rhodioloside	300.12	119.61	Passed	-6.5
Vaccinium myrtillus L.	Cyanidin 3-O-alpha-L-arabinopyranoside	419.1	171.37	Passed	-6.2
Vaccinium myrtillus L.	Delphinidin 3-alpha-L-arabinopyranoside	435.09	191.6	Failed	-6.25
Vaccinium macrocarpon	Undecanal	170.17	17.07	Passed	-5.02
Vaccinium ashei	Vaccihein A	378.1	131.75	Passed	-5.43
Vaccinium macrocarpon Aiton	alpha-Tocotrienol	424.33	29.46	Passed	-4.87
Vaccinium macrocarpon Aiton	beta-Tocotrienol	410.32	29.46	Passed	-4.76
Vaccinium macrocarpon Aiton	delta-Tocotrienol	396.3	29.46	Passed	-4.73

Vaccinium macrocarpon Aiton	gamma-Tocotrienol	410.32	29.46	Passed	-4.81
Vaccinium spp.	3-Phenylpropanol	136.09	20.23	Passed	-4.93
Vaccinium macrocarpon	Octadecan-2-one	268.28	17.07	Passed	-5.04
Vaccinium arboreum Marsh.	Delphinidin 3-O-galactoside	465.1	211.83	Failed	-6.46
Vaccinium arboreum Marsh.	Delphinidin 3-O-glucoside	465.1	211.83	Failed	-6.48
Vaccinium padifolium	Malvidin 3-O-glucoside	493.13	189.83	Failed	-6.15
Vaccinium oxycoccus	4-(4-Hydroxyphenyl)-2-butanone	164.08	37.3	Passed	-4.4
Vaccinium macrocarpon	Jaboticabin	334.07	133.52	Passed	-5.43
Vaccinium myrtillus	Isoprene	68.06	0	Passed	-4.54
Vaccinium oxycoccus	4'-Methoxyacetophenone	150.07	26.3	Passed	-4.49
Vaccinium macrocarpon	3-Pentanol	88.09	20.23	Passed	-4.8
Vaccinium macrocarpon	Benzeneboronic acid	122.05	40.46	Passed	-4.52
Vaccinium vacillans	2-O-Acetylarbutin	314.1	125.68	Passed	-6.2
Vaccinium vitis-idaea	2-O-trans-Caffeoylarbutin	434.12	166.14	Passed	-6.36
Vaccinium vitis-idaea	6-O-Acetylarbutin	314.1	125.68	Passed	-6.01
Vaccinium vitis-idaea	2-Methylbutyl 2-methylbutanoate	172.15	26.3	Passed	-4.7
Vaccinium macrocarpon	Myricetin 3-arabinofuranoside	450.08	210.51	Failed	-6.32
Vaccinium myrtillus	Myrtine	167.13	20.31	Passed	-4.67
Vaccinium vitis-idaea	1-O-trans-Cinnamoyl-beta-D-glucopyranose	312.12	116.45	Passed	-5.43
Vaccinium macrocarpon	zeta-Carotene	540.47	0	Failed	-4.75
Vaccinium angustifolium	Petunidin	317.07	121.68	Passed	-5.56
Vaccinium vitis-idaea, Vaccinium macrocarpon	2-O-Benzoyl-D-glucose	284.09	116.45	Passed	-5.81
Vaccinium vitis-idaea, Vaccinium oxycoccus	4-Hydroxy-2-oxobutanoic acid	118.03	74.6	Passed	-5.51
Vaccinium spp	D-Vacciniin	284.09	116.45	Passed	-5.93
Vaccinium vitis-idaea	3-hydroxy-2-oxobutanoic acid	118.03	74.6	Passed	-5.6
Vaccinium vitis-idaea	3,4,5-trihydroxy-6-(hydroxymethyl)oxan-2-yl (2E)-3-phenylprop-2-enoate	310.11	116.45	Passed	-5.72
Vaccinium vitis-idaea	Cyanidin 3-O-galactoside	449.11	191.6	Failed	-6.44
Vaccinium vitis-idaea	2-O-Caffeoylarbutin	434.12	166.14	Passed	-6.31
Vaccinium microcarpa	3,5,5-Trimethyl-2-cyclohexen-1-one	138.1	17.07	Passed	-4.66
Vaccinium spp	3-(2-propenoic acid)-o-benzoquinone	178.03	71.44	Passed	-4.97
Vaccinium spp	divinylchlorophyllide	612.22	115.25	Passed	-5.54
Vaccinium spp	pelargonidin 3-O-beta-D-p-coumaroylglucoside	578.14	196.35	Failed	-6.39
Vaccinium corymbosum	Malvidin 3-galactoside	493.13	189.83	Failed	-6.22
Vaccinium spp	Benzyl formate	136.05	26.3	Passed	-4.58
Vaccinium spp	N-Acetylglucosamine 6-phosphate	301.06	165.78	Passed	-5.68
Vaccinium spp	3-hydroxy-3-phenylpropionyl-CoA	915.17	390.84	Failed	-6.02
Vaccinium spp	delta7-Avenasterol	412.37	20.23	Passed	-4.94
Vaccinium spp	O-Acetylserine	147.05	89.62	Passed	-5.84
Vaccinium spp	5-Aminolevulinic acid	131.06	80.39	Passed	-5.38
